# Enhancer Extrachromosomal Circular DNA ANKRD28 Elicits Drug Resistance via POU2F2‐Mediated Transcriptional Network in Multiple Myeloma

**DOI:** 10.1002/advs.202415695

**Published:** 2025-04-01

**Authors:** Binzhen Chen, Jia Liu, Yaoxin Zhang, Changming Shi, Di Zhu, Guoqiang Zhang, Fei Xiao, Lu Zhong, Minyue Zhang, Lai Guan Ng, Honghui Huang, Tingting Lu, Jian Hou

**Affiliations:** ^1^ Department of Hematology Renji Hospital Shanghai Jiao Tong University School of Medicine Shanghai 200127 China; ^2^ Sheng Yushou Center of Cell Biology and Immunology Department of Genetics and Developmental Science School of Life Sciences and Biotechnology Shanghai Jiao Tong University Shanghai 200240 China; ^3^ Zhejiang Provincial Key Laboratory of Pancreatic Disease The First Affiliated Hospital Zhejiang University School of Medicine Hangzhou Zhejiang 310009 China; ^4^ National Center for Gene Research National Key Laboratory of Plant Molecular Genetics CAS Center for Excellence in Molecular Plant Sciences Institute of Plant Physiology and Ecology Chinese Academy of Sciences Shanghai 200032 China; ^5^ University of Chinese Academy of Sciences Beijing 100039 China; ^6^ Shanghai Immune Therapy Institute Renji Hospital Shanghai Jiao Tong University School of Medicine Shanghai 200127 China

**Keywords:** multiple myeloma, extrachromosomal circular DNA, drug resistance, enhancer, transcriptional regulation

## Abstract

Multiple myeloma (MM) remains an incurable disease primarily due to the emergence of drug resistance, and the underlying mechanisms remain unclear. Extrachromosomal circular DNAs (eccDNAs) are prevalent in cancer genomes of both coding and non‐coding regions. However, the role of non‐coding eccDNA regions that serve as enhancers has been largely overlooked. Here, genome‐wide profiling of serum eccDNAs from donors and MM patients who responded well or poorly to bortezomib‐lenalidomide‐dexamethasone (VRd) therapy is characterized. A high copy number of eccDNA ANKRD28 (eccANKRD28) predicts poor therapy response and prognosis but enhanced transcriptional activity. Established VRd‐resistant MM cell lines exhibit a higher abundance of eccANKRD28, and CRISPR/Cas9‐mediated elevation of eccANKRD28 desensitizes bortezomib and lenalidomide treatment both in vitro and in vivo. Integrated multi‐omics analysis (H3K27ac ChIP‐seq, scRNA‐seq, scATAC‐seq, CUT&Tag, et al.) identifies eccANKRD28 as an active enhancer involved in drug resistance driven by the key transcription factor, POU class 2 homeobox 2 (POU2F2). POU2F2 interacts with sequence‐specific eccANKRD28 as well as RUNX1 and RUNX2 motifs to form the protein complex, which activates the promoter of oncogenes, including *IRF4*, *JUNB*, *IKZF3*, *RUNX3, and BCL2*. This study elucidates the potential transcriptional network of enhancer eccANKRD28 in MM drug resistance from a previously unrecognized epigenetic perspective.

## Graphical abstract

Enhancer eccANKRD28‐manipulated MM cells have been demonstrated to facilitate drug resistance and promote MM progression by activating the key transcription factor, POU2F2. POU2F2 interacts with sequence‐specific eccANKRD28 as well as RUNX1 and RUNX2 motifs to form the protein complex, which activates the promoter of oncogenes (IRF4, JUNB, IKZF3, et al.) both in vitro and in vivo.

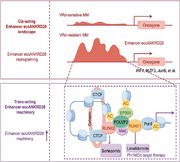

## 1 Introduction

The current therapeutic approach for newly diagnosed multiple myeloma (NDMM) utilizing proteasome inhibitors (PI) and immunomodulatory drugs (IMiDs), especially the most commonly used bortezomib‐lenalidomide‐dexamethasone (VRd) targeted therapy, has shown efficacy in achieving deep remission,^[^ ^1^ ^]^ yet most patients eventually develop resistance.^[^ ^2^ ^]^ This drug resistance is closely associated with the intricate epigenomic landscape of MM.^[^ ^3^ ^]^ Therefore, the mechanism that induces VRd resistance in MM and identifying novel targets are urgently needed.

Extrachromosomal circular DNAs (eccDNAs) are circular DNA molecules derived from chromosomes but exist as separate entities from chromosomal DNA upon formation.^[^ ^4^ ^]^ A comprehensive pan‐cancer analysis of ATAC‐sequencing libraries from 23 tumor types identified over 18000 eccDNAs, many of which harbor well‐known cancer‐promoting genes.^[^ ^5^ ^]^ EccDNA has already been found in both circulating and hematopoietic cells and has been suggested to reflect disease status and promote malignant phenotypes.^[^ ^6^, ^7^ ^]^ EccDNA can contain entire gene sequences, specific oncogenes with amplified copy numbers, and heightened transcription levels. The upregulation of oncogenes by eccDNA is implicated in tumor growth.^[^ ^8^ ^]^ EccDNAs containing oncogenes can drive intratumoral heterogeneity and contribute to extreme copy number amplification through uneven segregation. A recent study demonstrated that approximately half of the amplifications in MM can be attributed to eccDNA formation, highlighting the intersection of MM genomics and epigenetics.^[^ ^9^ ^]^ In particular, microDNA, eccDNAs <1000 bp in size derived from chromosomal genomic sequences, is released from normal and tumor cells into the circulation and is often more stable than linear cell‐free DNA (cfDNA).^[^ ^10^ ^]^ The precise contribution of microDNA, particularly non‐coding DNA elements that do not contain open reading frames (ORFs), to resistance mechanisms remains inadequately elucidated, with little experimental evidence currently available to establish their oncogenic and epigenetic roles definitively.

Ankyrin repeat domain 28 (*ANKRD28*) has been confirmed as an oncogene in acute myeloid leukemia (AML).^[^ ^11^, ^12^ ^]^ Analogously, super‐enhancers‐regulated *ANKRD28* correlates with poor prognosis in patients with germinal center B‐cell diffuse large B‐cell lymphoma (GCB‐DLBCL).^[^ ^13^ ^]^ Despite the evidence in other hematologic neoplasms, the role of *ANKRD28*, especially its non‐coding DNA regions that served as enhancers, remains unclear in MM. This study elucidated the crucial role of serum eccANKRD28 extrachromosomal amplification in facilitating drug resistance, indicating a novel extracellular nucleic acid biomarker for assessing therapy response.

## 2 Results

### 2.1 Genome‐Wide Characterization of Serum EccDNAs in Multiple Myeloma

**Figure 1:**
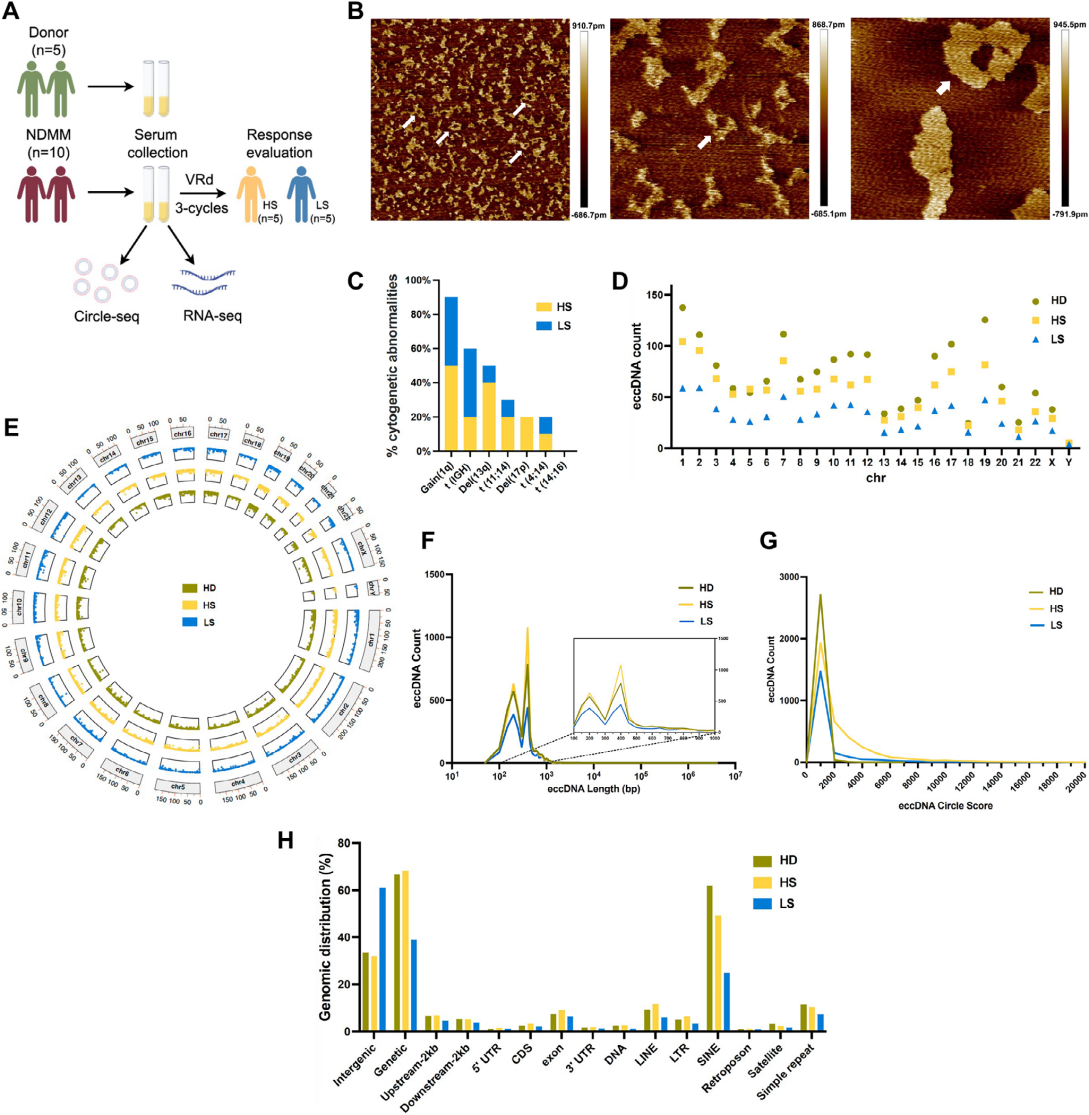
Genome‐wide characterization of serum eccDNAs in multiple myeloma. A) Flowchart illustrating sample collection process within the study design. B) AFM images of extracted eccDNAs in MM patient's serum. C) Stack bar chart of the most frequent genomic events in HS and LS samples, broken down by cytogenetic group. D) EccDNA frequency relative to chromosomes. EccDNA counts per chromosome in healthy donors (HD, green circle), high‐sensitivity individuals (HS, achieving sCR/CR after three cycles of VRd, yellow square), and low‐sensitivity individuals (LS, achieving PR/MR/SD/PD after three cycles of VRd, blue triangle). n = 5 in each group. E) Circos plot showing the chromosomal distribution of eccDNAs. The four circles in the plot, from outermost to innermost, represent the human chromosomes and the distributions of eccDNAs in the HD (green), HS (yellow), and LS (blue) samples. F) Length distribution of eccDNAs in the three groups, with significant peaks observed at 100 and 1000 bases. G) Circle scores of eccDNAs in the HD (green), HS (yellow), and LS (blue) groups. H) Genomic distributions of eccDNAs in the HD (green), HS (yellow), and LS (blue) groups. Abbreviations: sCR, stringent complete response; CR, complete response; PR, partial response; MR, minimal response; SD, stable disease; PD, progressive disease.

To investigate serum eccDNA from NDMM patients on a genomic scale, we employed Circle‐seq and RNA‐seq. Serum samples were collected before administering three cycles of the VRd regimen, followed by evaluating therapeutic responses (**Figure** **1**A). EccDNAs were initially isolated from the serum of three distinct groups: healthy donors (HD, n = 5), individuals with high sensitivity (HS, n = 5), and those with low sensitivity (LS, n = 5) to VRd. To validate the existence of eccDNAs in the patient's serum, atomic force microscopy (AFM) was used to visualize the circular structure of eccDNAs (Figure 1B). Subsequently, the chromosomal origins of eccDNAs were analyzed through gene annotation. In total, 19218 genes were identified, 6343 eccDNAs were mapped to genic regions, and 52.25% of eccDNAs overlapped with intergenic regions (Figure S1A, Supporting Information).

The formation of eccDNA reflects genomic instability, with focal amplification often resulting from the fusion of rearranged DNA segments from different chromosomal locations. To further test this assertion, we examined whether eccDNA preferentially originates from chromosomal abnormalities, including IgH translocations such as t (4;14), t (11;14), and t (14;16), as well as other mutations like del(17p) and gain(1q). These alterations contribute to MM progression and are, in some cases, associated with treatment resistance.^[^ ^14^, ^15^ ^]^ Among these 10 MM patients, 1q gain was the most common adverse chromosomal aberration (Figure 1C). We analyzed the chromosomal distribution of average eccDNA frequency to gain further insight. Interestingly, chr1 exhibited the highest average count of eccDNAs compared to other chromosomes, surpassing even the gene‐rich chr19, which has been identified as significant in different cancer types (Figure 1D).^[^ ^16^ ^]^ Paired‐end reads were aligned to chromosomes to characterize eccDNA and detect structural variations resulting from DNA circularization (Figure 1E). Consistent with previous observations, eccDNAs were distributed strikingly consistently between donors and MM samples.

Alterations in fragmentation patterns reflect changes in the chromatin structure before the release of cfDNA, as well as other genomic and epigenomic alterations occurring during tumorigenesis. We subsequently analyzed the unique structural characteristics of these 15 sequencing samples, focusing on their eccDNA length, circle score, and genomic distribution. We observed that the average length of eccDNA was ranked in descending order: HD > HS > LS, with a statistically significant difference observed between the HD and LS groups (P<0.05, Figure S1B, Supporting Information). It can be observed that the distribution characteristics of eccDNA length are very similar to those of cfDNA length (shorter in cancer patients and longer in healthy individuals).^[^ ^17^, ^18^ ^]^ Here, the eccDNA identified using the Circle‐seq method ranged in length from 10 bp to 10000 kb, with the majority falling between 100 to 1000 bp, suggesting that shorter eccDNAs are more prevalent in serum (Figure 1F). The circle score, a scoring scheme that considers alignment quality, split segment length, and the number of split reads supporting circular DNA, was utilized to evaluate the quality of eccDNA among the three groups.^[^ ^19^ ^]^ We observed that the proportion of eccDNA in the HD group was more significant than that in the HS group and that the LS group was the lowest at the same circle score level. As the circle score increased, the proportion of eccDNA initially increased but subsequently decreased. Specifically, within the lower range of the Circle score (≈0–1000), an increase in detected eccDNAs correlates with an increase in the Circle score. Conversely, when the circle score exceeded 1000, the proportion of eccDNA in all the samples continuously decreased to markedly low levels (Figure 1G). To explore the possible origin of eccDNAs, we mapped the overall population of serum eccDNAs to different classes of genomic elements via tagmentation. Non‐coding sequences, including intronic and intergenic regions, often constitute enhancers, and compared with nontumor samples, eccDNAs in tumors present a more significant proportion of cis‐acting elements, particularly enhancers.^[^ ^20^, ^21^ ^]^ Notably, eccDNAs were significantly enriched in intergenic and genetic regions and short interspersed elements (SINEs) (Figure 1H), suggesting that these regions, rather than gene‐rich areas, were more likely to generate eccDNAs. Furthermore, in the intergenic region, the distribution of eccDNA in the LS group was significantly greater than that in both the HD and HS groups, indicating that the development of therapeutic response in the LS group may be correlated with enhancer activity, which is often affected by genetic perturbations and epigenetic changes.

### 2.2 Differential EccDNAs and EccGenes between HS and LS Patients

**Figure 2:**
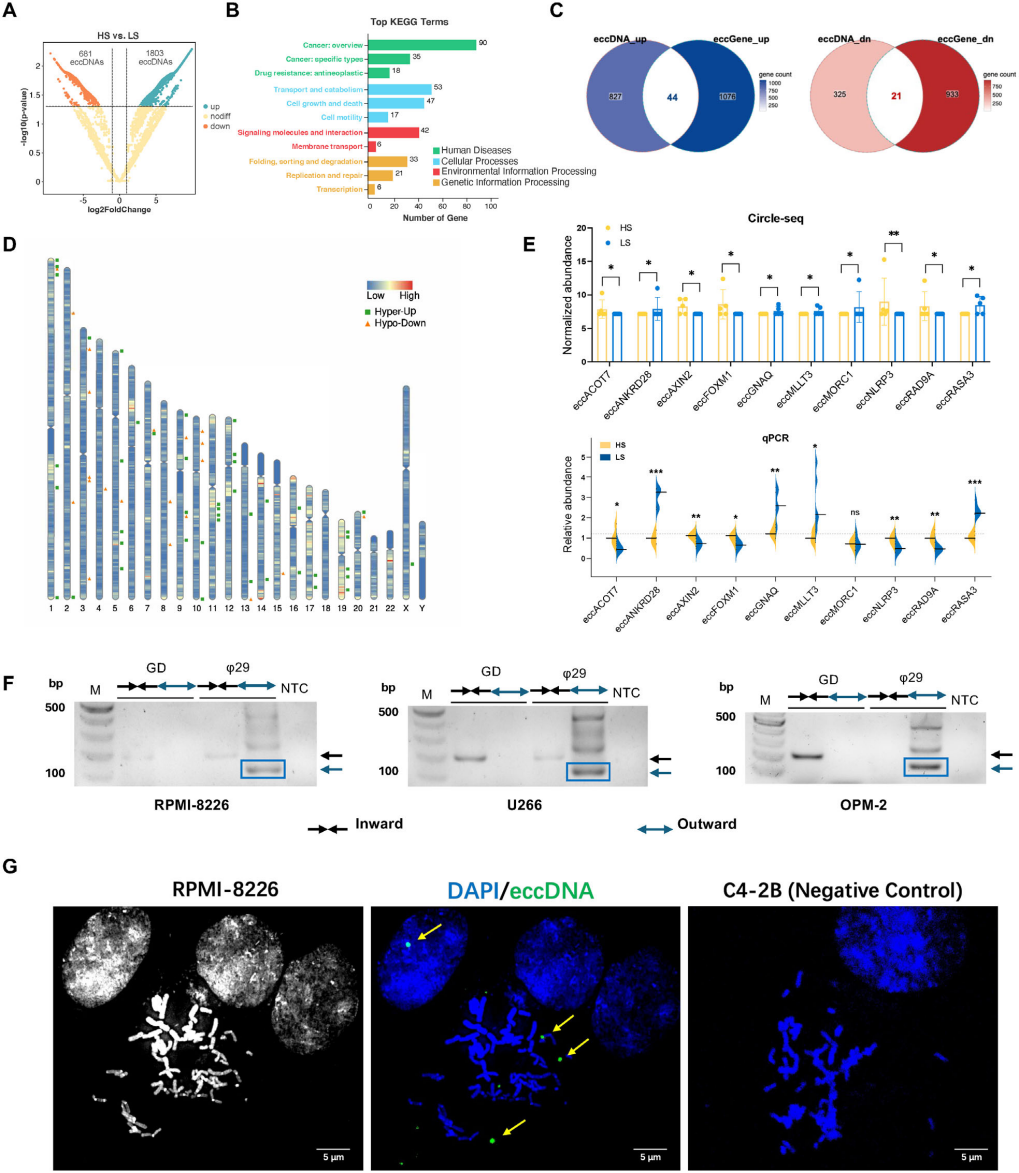
Differential eccDNAs and eccGenes between HS and LS patients. A) Volcano plot of differential eccDNAs detected between HS and LS patients. The upregulated and downregulated eccDNAs in HS and LS patients are marked in green and red, respectively (Log2FC > 2, P value < 0.05). B) KEGG pathway analysis of total differential eccGenes in NDMM patients. C) Venn diagrams showing the overlap of all differential eccGenes and eccDNAs. Left, upregulated targets in both sequences (blue); Right, downregulated targets in both sequences (red). D) Chromosomal distribution of 44 hyper‐up (green square) and 21 hypo‐down (orange circle) eccDNAs. The color intensity indicates differences in eccDNA number. E) Bar plot and split violin plot for qPCR validation of the differences in normalized counts of 10 candidate eccDNA Circle‐seq cohorts and another 5 HS (yellow) and 5 LS (blue) samples. ^*^ *P* < 0.05; ^**^ *P* < 0.01; ^***^ *P* < 0.001. F) Outward and inward PCR detection of eccANKRD28 in RPMI‐8226, U266, and OPM‐2 cells. The DNA bands marked with blue boxes were gel‐purified and sequenced. G) Detection of eccANKRD28 in the RPMI‐8226 cell line (gray, left) by FISH: metaphase spread of chromosome (blue) was stained with a probe (green) against the eccANKRD28 locus chr3:15768491‐15768703 (middle). C4‐2B (right) is the negative control cell line, for its spread does not have an extrachromosomal DNA signal. Yellow arrows mark the eccDNA signals.

Furthermore, we sought to investigate whether there were differences in the content of eccDNAs between HS and LS patients. 2484 eccDNAs were preliminarily screened with a threshold of Log2FC >2 and adjusted p < 0.05. Compared with those in the LS patients, 1803 eccDNAs were upregulated, and 681 eccDNAs were downregulated in the HS patients (**Figure** **2**A). Furthermore, KEGG enrichment analyses were performed based on the differential eccDNA‐overlapping genes (eccGenes). Differential eccGenes between the HS and LS groups were enriched predominantly in processes such as human disease terms (cancer and antineoplastic drug resistance) and genetic information processing terms (replication, repair, and transcription) (Figure 2B). Among these genes, 44 eccDNAs were co‐upregulated (hyper‐upregulated), and 21 were co‐downregulated (hypo‐downregulated) with eccGenes (Figure 2C). The locations of eccDNA shedding indicate specific chromosomal regions excised from linear chromosomes and subsequently circularized to form eccDNAs.^[^ ^22^ ^]^ We focused on 18 hyper‐up and 9 hypo‐down eccDNAs with eccGenes enriched in cancer‐ or myeloma‐related processes. These 27 eccDNAs revealed more shedding locations in LS patients, particularly those enriched on chromosome 3. Conversely, HS samples exhibited more eccDNA shedding locations on chromosomes 1, 5, 11, and 19 (Figure 2D). Then, we selected 10 eccDNA candidates by higher‐ranking Log2FC and restricted the Circle‐seq signal by considering only those that were detectable in more than half of the samples. To further validate our sequencing results, qPCR and outward PCR were performed in another 10 NDMM cohorts (Figure S2A,B, Supporting Information). Among them, the abundance of eccANKRD28 (chr3:15768491‐15768703, 213 bp) exhibited statistically significant differences according to both Circle‐seq and qPCR analysis (Figure 2E). We also intersected eccDNAs and eccGenes that were co‐regulated between the HS and LS groups and between the HS+LS and HD groups, resulting in two candidate eccDNAs (eccANKRD28 and eccMORC1, Figure S2C, Supporting Information). Compared with those in the HS group, the proportions of eccANKRD28 and eccMORC1 were both more significant in the LS group, and relative to those in the HD group, they were also both greater in the MM group. In contrast, eccMORC1 did not show a statistically significant difference between groups in the validation cohort in Figure 2E, indicating that eccANKRD28 is involved in both the pathogenesis of MM and VRd resistance. Based on these integrative analyses, eccANKRD28 was chosen for further investigation.

To verify the specific circular structure of eccANKRD28, inward PCR and outward PCR were conducted in the RPMI‐8226, U266, and OPM‐2 cell lines (Figure 2F), with specific primers designed to target the junction sites of each candidate. The elimination of linear DNA was validated through PCR analysis of the *COX5B* gene, which was found to be absent in eccDNA, as previously reported (Figure S2D, Supporting Information).^[^ ^23^ ^]^ The results revealed that eccANKRD28 was not present in the genomic DNA. The outward PCR products were utilized for Sanger sequencing to identify the junction sites of the eccDNA (Figure S2E, Supporting Information). To confirm its existence, we also performed metaphase FISH using junction‐specific probes in RPMI‐8226 cells compared with C4‐2B (prostate cancer cell line) as a negative control validated in CytoCellDB (Figure 2G).^[^ ^24^ ^]^ For C4‐2B, the spreads do not show an eccANKRD28 signal. The positive signals in RPMI‐8226 representing eccANKRD28 were mainly observed off the chromosomes, confirming the extrachromosomal feature of eccANKRD28.

### 2.3 Potential Role of eccANKRD28 in the Prediction and Prognostic Stratification of NDMM Patients

**Figure 3:**
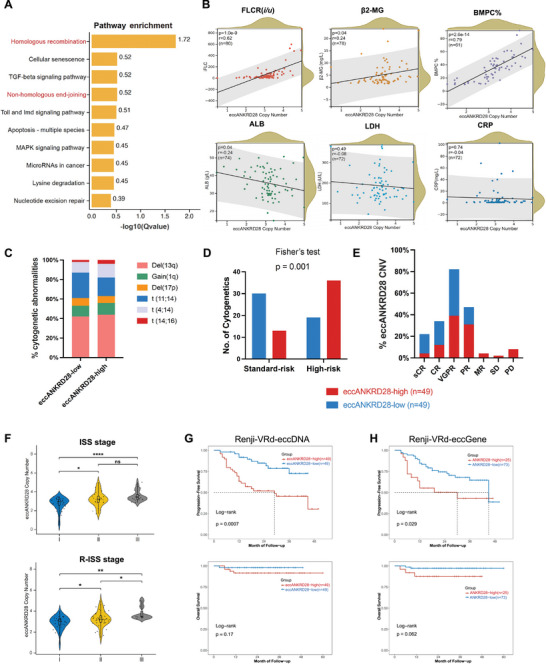
Potential role of eccANKRD28 in the prediction and prognostic stratification of NDMM patients. A) Pathway enrichment of all differential eccGenes. B) eccANKRD28 copy number across junction sites was measured by qPCR. Spearman correlation analysis of the serum free light chain ratios of involved/uninvolved [FLCR(i/u)], β2‐microglobulin (β2‐MG), the percentage of bone marrow plasma cells (BMPC%), albumin (ALB), lactic dehydrogenase (LDH) and C‐reactive protein (CRP) with the serum eccANKRD28 copy number in 98 NDMM patients in the Renji‐VRd cohort. Each dot represents an independent individual. The gray shaded area represents the 95% CI. The Spearman correlation coefficient (r), P value (p), and sample number (n) are indicated. C) Stack bar chart of chromosomal abnormality events between the eccANKRD28‐high and eccANKRD28‐low groups, broken down by cytogenetic group. D) Bar plot reporting the number of high‐ and standard‐risk cytogenetics subgroups between the eccANKRD28‐high (red) and eccANKRD28‐low (blue) (p = 0.001, Fisher's exact test). The high‐risk was defined as the presence of at least one of the following: t(4;14), t(14;16), del(17p), and amp(1q). E) Stack bar chart of VRd responses between the eccANKRD28‐high (red) and eccANKRD28‐low (blue) groups. F) ISS stage and R‐ISS stage correlation with eccANKRD28 copy number. ^*^ *P* < 0.05; ^**^ *P* < 0.01; ^****^ *P* < 0.0001. G) Kaplan–Meier curves depicting PFS (p = 0.0007) and OS (p = 0.17) in the 98 NDMM patients in the Renji‐VRd cohort stratified by the copy number of eccANKRD28. H) Kaplan–Meier curves of PFS (p = 0.029) and OS (p = 0.062) in the 98 NDMM patients in the Renji‐VRd cohort stratified by eccGene *ANKRD28*. P values were derived from a two‐sided log‐rank test without correction for multiple hypotheses.

In response to selective stress, such as carcinogenesis and drug resistance during cancer treatment, the copy number of eccDNA can change dynamically.^[^ ^4^ ^]^ To determine the origin of serum eccANKRD28, the fluorescence intensity in five CD138+ cells and paired CD138‐ cells was determined via FISH. We found that CD138+ MM cells had a much greater intensity of eccANKRD28 than other cells did, indicating that serum eccANKRD28 was derived mainly from CD138+ plasma cells in the bone marrow (Figure S3A,B, Supporting Information). We conducted pathway enrichment analysis on the genes overlapping with highly abundant eccDNA in the LS group, revealing their enrichment in pathways related to canonical homologous recombination (cHR) and non‐homologous end joining (NHEJ) (**Figure** **3**A), suggesting that the functions of cHR and NHEJ are correlated with increased eccDNA levels and amplified oncogene copy numbers within tumor cells, resulting in resistance to chemotherapy.

To elucidate the correlation between eccANKRD28 and myeloma status, we investigated the correlation between eccANKRD28 CNV and the clinical characteristics of the serum of NDMM patients. The detection of circular DNA in liquid biopsies is potentially helpful in identifying tumorigenesis and progression; however, several difficulties still exist. Most importantly, reliable methods for quantifying the abundance of circular DNA are lacking.^[^ ^25^ ^]^ Thus, we validated the abundance of eccANKRD28 through copy number variation (CNV) quantification via qPCR. Among the parameters, eccANKRD28 CNV was positively correlated with the involved/uninvolved free light chain ratio [FLCR(i/u)] (p = 1.0e‐9, r = 0.62, n = 80), β2‐MG (p = 0.04, r = 0.24, n = 78), and bone marrow plasma cell percentage (BMPC%) (p = 2.6e‐14, r = 0.79, n = 61), where FLCR(i/u) and BMPC% are both key parameters used to assess the tumor burden in myeloma. Notably, eccANKRD28 CNV was negatively correlated with ALB (p = 0.04, r = ‐0.24, n = 74) (Figure 3B). The distribution of chromosomal abnormalities varied little between the eccANKRD28‐high and eccANKRD28‐low groups (Figure 3C). However, there were significant differences between high‐ and standard‐risk cytogenetics subgroups (p = 0.001) (Figure 3D). Furthermore, the distribution among serial VRd responses also differed significantly between the low‐ and high‐eccANKRD28 groups. Most notably, patients who achieved MR/SD/PD were exclusively in the eccANKRD28‐high group (Figure 3E). Next, we explored the potential application of eccANKRD28 CNVs for the prognostic stratification of NDMM. Higher ISS and R‐ISS stages predicted relapse and mortality. The eccANKRD28 CNV relatively increased with increasing ISS and R‐ISS stages (Figure 3F), indicating the broad involvement of eccANKRD28 amplification in predicting treatment response.

The amplification of eccDNA‐based oncogenes is associated with aggressive cancer behavior, including tumor growth, metastasis, and drug resistance, resulting in poor outcomes in cancer. Thus, we reinforced the above observations in an NDMM cohort, including 98 patients assigned to the VRd regimen at Renji Hospital from Jan 5, 2018, to Dec 28, 2023. At a median follow‐up of 30 months (IQR 8–60 m), patients with high eccANKRD28 copy numbers at diagnosis had significantly poor PFS (median PFS 29 months versus not reached), with a striking hazard ratio (HR) of 3.14 (95% CI 1.64–5.99, p = 0.0007). The median overall survival (OS) was not reached in either group (HR = 4.06, 95% CI 0.70–23.41; p = 0.17; Figure 3G). Similarly, we also found that serum *ANKRD28*, the eccGene amplified from eccANKRD28, was inversely correlated with poor PFS (median PFS 30 months versus 45 months, HR = 2.06, 95% CI 0.93–4.54; p = 0.029) in the Renji‐VRd cohort. The eccGene corresponding median OS was not reached in either group (HR = 4.68, 95% CI 0.61–36.12; p = 0.062; Figure 3H). Then we collected 5 CD138+ BMPC samples from the Renji‐VRd cohort and found that eccANKRD28 was not present in the genomic DNA of MM patients (Figure S3C, Supporting Information). Therefore, we used the CoMMpass‐VRd data to further support the conclusions from our center work, described above (Figure S3D, Supporting Information). Our findings suggest that the serum eccANKRD28 level could serve as a risk factor and a promising marker for monitoring MM progression.

### 2.4 EccANKRD28 Dynamics Correlate with the Development of VRd Resistance and Amplify Transcription Activity

**Figure 4:**
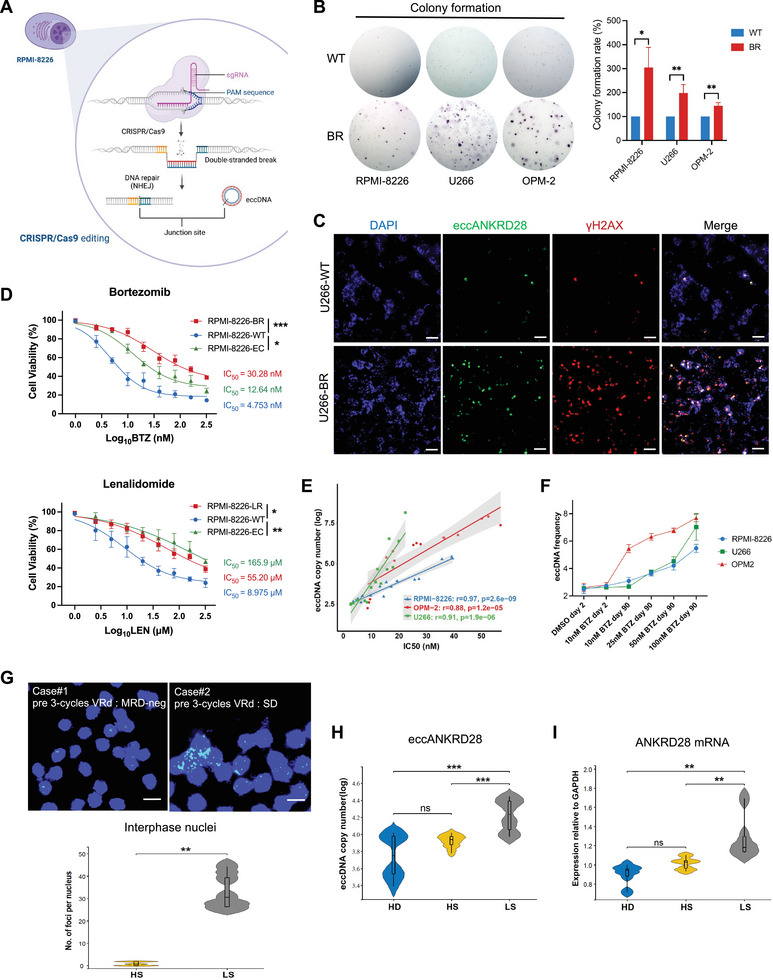
EccANKRD28 dynamics are correlated with the development of VRd resistance and amplify transcription activity. A) CRISPR/Cas9‐mediated generation of custom eccDNA in RPMI‐8226 cells. Abbreviations: CRISPR/Cas9, clustered regularly interspaced short palindromic repeats; NHEJ, non‐homologous end joining. The graphical scheme was created with BioRender. B) Left: The representative colony formation images of RPMI‐8226, U266, and OPM‐2 cells between WT and BR groups. Right: Colony formation rates were expressed as the percentage of colonies in BR cultures compared with that in WT cultures. Each assay was performed in triplicate. C) Representative dual immunofluorescence‐FISH images for eccANKRD28 (green) and γH2AX (red) protein expression in U266 wild‐type (WT) and bortezomib‐resistant (BR) cells. Scale bar, 10 µm. D) Dose‒response curves for the RPMI‐8226 wild‐type (WT), RPMI‐8226 bortezomib‐ or lenalidomide‐resistant (BR or LR) and RPMI‐8226 eccDNA‐CRISPR/Cas9 (EC) cell lines used to determine sensitivity to bortezomib and lenalidomide. The cells were incubated with bortezomib and lenalidomide at serial dilutions, starting at doses of 5 nm bortezomib and 5 µm lenalidomide, for 48 h before cell viability was determined via a CCK‐8 assay. Results are shown as the mean ± SD of three independent experiments. Statistical significance was assessed using two‐way ANOVA (^*^ *P* < 0.05; ^**^ *P* < 0.01; ^***^ *P* < 0.001). E) Multiple correlation analysis between the IC_50_ and eccANKRD28 copy number in RPMI‐8226, U266, and OPM‐2 cells. F) Mean eccANKRD28 copy number after eccANKRD28 induction on day 0 ± BTZ treatment beginning on day 3. G) Interphase FISH microscopy for eccANKRD28. Case #1 and Case #2 were representative images of CD138+ sorted plasma cells from NDMM, Case #1 was evaluated as MRD‐neg (HS group, n = 5), and Case #2 was evaluated as SD (LS group, n = 5) after three cycles of the VRd regimen. Representative images from a series of 10 images of each subject. Scale bar, 10 µm. H‐I) Copy number of eccANKRD28 and ANKRD28 mRNA expression determined by qPCR in the HD/HS/LS group. ^**^ *P* < 0.01; ^***^ *P* < 0.001.

To further identify the key role of eccANKRD28 dynamics in VRd‐related therapy resistance, we aimed to quantify the evolution of eccANKRD28 from its inception experimentally. We chose CRISPR technology to generate endogenous eccANKRD28 in RPMI‐8226 cells (**Figure** **4**A), which we term RPMI‐8226‐EC.^[^ ^26^ ^]^ We utilized lentiviral vectors to introduce Cas9 and single‐guide RNAs (sgRNAs) into cells and subsequently measured cell proliferation and eccDNA copy number. The transfection efficiency evaluated with mCherry+ cells was ≈80%, and the eccDNA copy number was calculated via a qPCR standard curve (Figure S4A, Supporting Information). In addition, using parental cells (RPMI‐8226‐WT), we constructed bortezomib‐resistant (RPMI‐8226‐BR) and lenalidomide‐resistant (RPMI‐8226‐LR) cells, for which the IC_50_ of the drug was increased by at least 4 fold (Figure S4B, Supporting Information). The colony formation capacity (Figure 4B) was markedly enhanced. The γH2AX is a specific biomarker for DNA double‐strand breaks (DSBs). DSBs are the most deleterious DNA damage that contributes to genomic instability and increases tumorigenesis risk. Consequently, dysregulation of γH2AX is associated with human cancer. To elucidate the role of eccANKRD28 in MM and its relationship with γH2AX, we investigated the impact of elevated eccANKRD28 levels on the drug resistance of MM cells. As shown in Figure 4C, bortezomib resistance led to an increase in the number of residual γH2AX foci. On the other hand, γH2AX was also a key regulator of the DNA repair system after DSBs, thereby contributing to drug resistance of MM cells. This also indicated that the changes in eccANKRD28 abundance in MM‐resistant cell lines were due to endogenous DNA damage. As expected, the eccANKRD28 copy number was successfully increased via CRISPR/Cas9 editing. Compared to RPMI‐8226‐WT cells, the IC_50_ values were significantly increased in both RPMI‐8226‐BR and RPMI‐8226‐LR cells. Furthermore, CRISPR/Cas9 editing of eccANKRD28 desensitized parental RPMI‐8226 cells to bortezomib and lenalidomide treatment (Figure 4D). Correlative analyses of eccANKRD28 copy numbers and IC_50_ values demonstrated high concordance with our models (Figure 4E), and we observed a substantial, dose‐dependent increase in eccANKRD28 copy number in response to bortezomib treatment (Figure 4F), which suggested strong positive selection for oncogene‐bearing eccANKRD28 in myeloma.

Interestingly, DNA FISH for eccANKRD28 in the nuclei of CD138+ BMPCs from patients with NDMM confirmed this finding (Figure 4G). In interphase nuclei, signal intensity analysis indicated that eccANKRD28 signal intensity was preferentially located toward the nuclear periphery. Interestingly, when 5 HDs were compared with 10 HS and LS patients, the eccANKRD28 copy number was significantly correlated with the VRd‐low sensitivity. Still, it was not significantly associated with the VRd‐high sensitivity. Additionally, the eccANKRD28 DNA signal pattern was confirmed in both BMPCs. Moreover, the eccANKRD28 copy number was positively correlated with therapy response, accompanied by increased transcriptional activity (Figure 4H,I). Taken together, both the cell line and patient data agreed with models of eccDNA, along with their oncogenic content, which were subjected to significant selective pressure, affecting the mean eccDNA‐containing oncogene amplification.

### 2.5 H3K27ac Enrichment Visualization and Enhancer EccANKRD28 Validation

**Figure 5:**
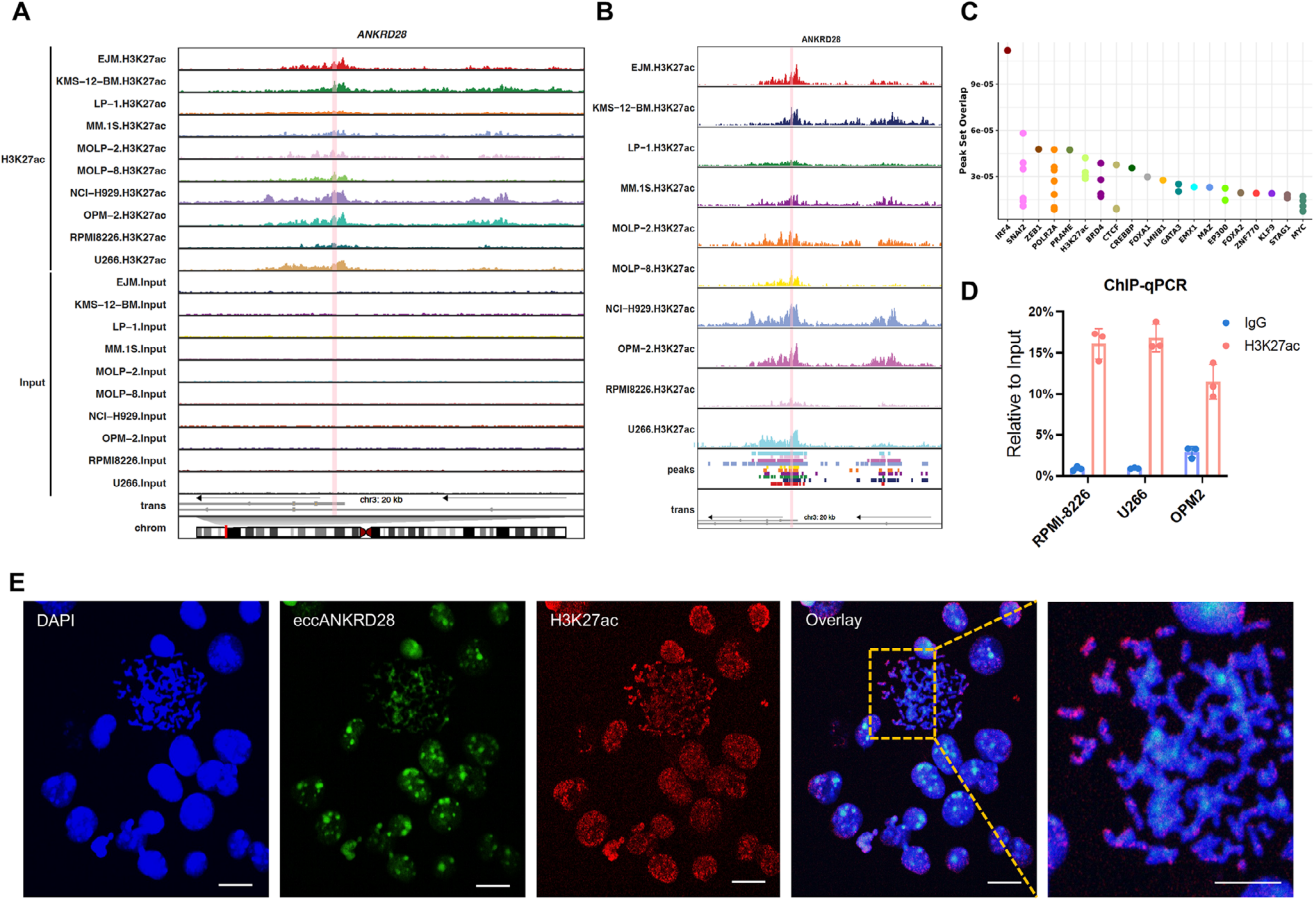
H3K27ac enrichment visualization and enhancer eccANKRD28 validation. A) H3K27ac ChIP‐seq track for signals in the ANKRD28 gene region of skew across 10 MMCLs (EJM, KMS‐12, LP‐1, MM.1S, MOLP‐2, MOLP‐8, NCI‐H929, OPM‐2, RPMI‐8226, and U266). B) Zoomed‐in image of the H3K27ac signal for eccANKRD28. The region (pink) in (A) and (B) is highlighted for the eccANKRD28 interval. C) Predicted factors bound in the eccANKRD28 interval via the Cistrome Data Browser. D) ChIP‒qPCR validation of eccANKRD28 binding with the H3K27ac protein in RPMI‐8226, U266, and OPM‐2 cells. E) Confocal dual immunofluorescence‐FISH microscopy of eccANKRD28 (green) and H3K27ac (red) colocalization in U266 metaphase cells. Box (yellow) is magnified (right). Scale bar, 5 µm.

Recent findings have indicated that enhancer‐containing eccDNAs may function as mobile regulatory elements for chromosomal transcription.^[^ ^27^, ^28^ ^]^ The enhancer activity promotes oncogene expression and facilitates chromosomal structural rearrangements. Since the chromosome region is entirely non‐coding, we mapped the active regulatory elements via H3K27ac ChIP‐seq. We identified strong H3K27ac signals directly within the *ANKRD28* region of skew across 10 MMCLs (**Figure** **5**A,B). Multiple lines of evidence support their functional activity: the enhancer eccANKRD28 interval chr3:15768491–15768703 is predicted to have significant binding overlap with transcription factors, including H3K27ac on the Cistrome DB (http://cistrome.org/db/#/) and ENCODE screen (Figure 5C; Figure S5A, Supporting Information).

Furthermore, ChIP‒qPCR revealed that eccANKRD28 could directly bind to H3K27ac in three different MMCLs (Figure 5D; Figure S5B,C, Supporting Information). Next, we assessed whether eccDNA foci colocalize within high local concentrations of the transcriptional machinery to create transcription hubs. Using dual immunofluorescence‐FISH, a distinct H3K27ac highly enriched signal was observed at the eccANKRD28 locus (Figure 5E). This observation provided the foundation to investigate the function of eccANKRD28 in gene regulation from an epigenetic perspective.

### 2.6 Enhancer EccANKRD28 Further Boosts VRd Resistance via Interaction with the POU and RUNX Family in RRMM

**Figure 6:**
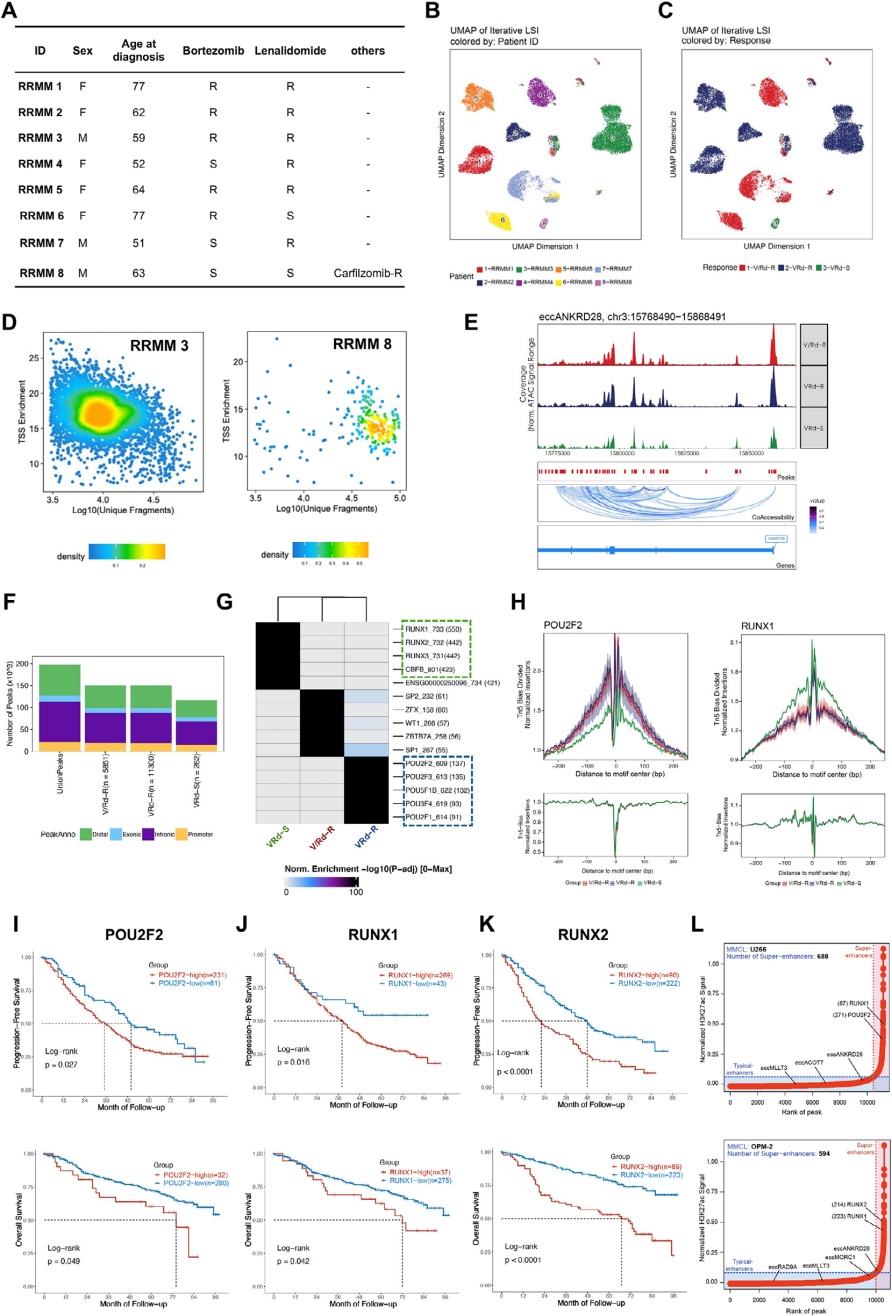
Enhancer eccANKRD28 promotes VRd resistance via interaction with the POU and RUNX family. A) Characteristics for relapsed/refractory multiple myeloma (RRMM) subjects available in both scRNA‐seq and scATAC‐seq. Patient IDs in this manuscript are renumbered with IDs used in Tirier et al.^[^ ^3^ ^]^ Before treatment, drugs against which a patient has been refractory are marked with an R while marked with an S if sensitive. B,C) Uniform manifold approximation and projection (UMAP) projection of cell clusters detected in 8 RRMMs of paired scATAC‐seq and scRNA‐seq data. Cell clusters are labeled based on patient ID in (B) and VRd response in (C). D) QC filtering plot for cells of RRMM3 and RRMM8 showing the TSS enrichment score versus unique nuclear fragments per cell. The dot color represents the density in AU of points in the plot. Dashed lines represent the filters for high‐quality single‐cell data (3000 unique nuclear fragments and TSS score greater than or equal to 7). E) Co‐accessibility shows eccANKRD28 track coverage identified by the scATAC‐seq signal by different responses. F) Bar plot showing the number of final peaks identified across patients with different VRd responses from the scATAC‐seq dataset. Bars are colored based on the location of the peak in the promoter, distal, exonic, or intronic regions. G) ComplexHeatmap enrichment for motifs enriched in scATAC‐seq peaks up‐ or downregulated in different VRd responses. H) TF footprint plot of POU2F2 and RUNX1 by different VRd responses. The red line represents the V/Rd‐R, the blue line represents the VRd‐R, and the green line represents the VRd‐S group. I–K) Kaplan–Meier plot for PFS and OS comparing 312 patients in the CoMMpass‐VRd cohort showing increased POU2F2 expression (I) (*P* = 0.027 for PFS and *P* = 0.047 for OS), increased RUNX1 expression (J) (*P* = 0.018 for PFS and *P* = 0.062 for OS). Increased RUNX2 expression (K) (*P* < 0.0001 for PFS and *P* < 0.0001 for OS) was associated with poor survival in MM patients. The log‐rank test evaluated significance. L) ROSE calling via H3K27ac ChIP‐seq revealed super‐enhancers (SEs, red) and typical enhancers (TEs, blue) in U266 and OPM‐2 cells (another 8 MMCLs in Figure S7A, Supporting Information). SEs were determined by the inflection point of the plot, VRd resistance‐associated SE‐TFs were annotated along the vertical axis, and VRd resistance‐eccDNAs were annotated along the horizontal axis. Box and whisker plots displaying the FPKMs for VRd resistance‐associated TF genes between SEs and TEs. Abbreviations: VRd‐R, bortezomib and lenalidomide double resistant; V/Rd‐R, either bortezomib or lenalidomide resistant; VRd‐S, bortezomib and lenalidomide double sensitive.

In eukaryotic genomes, the fundamental function of gene regulatory elements is the modulation of chromatin accessibility to facilitate the binding of transcription factors (TFs) and RNA polymerase. To identify the key TF mediating the activity of enhancer eccDNA, we integrated scATAC‐seq and scRNA‐seq datasets from 8 RRMM patients (**Figure** **6**A–C). In agreement with previous reports,^[^ ^29^ ^]^ we found that the enhancer eccANKRD28 exhibited increased chromatin accessibility (open chromatin) and ultralong‐range chromatin contact, as measured by scATAC‐seq, demonstrating the correlation between the presence of numerous chromatin open sites and the effectiveness of targeted drugs (Figure 6D,E). A greater extent of coverage and larger chromatin open areas are associated with increased transcriptional activity in cells. Moreover, analysis of the genome‐wide distribution of cis‐regulatory elements showed that the three VRd response groups were all located mainly within the intronic regions, unlike those previously reported,^[^ ^10^ ^]^ which contain sequences of exons that form si‐like RNAs to repress the endogenous gene (Figure 6F). To understand whether different groups of peaks were enriched for binding sites of specific TFs, we performed motif enrichment mapping to the ENCODE consortium. Notably, the RUNX family (RUNX1/RUNX2/RUNX3) and CBFβ, the RUNX1/3‐binding partner,^[^ ^30^ ^]^ were enriched mainly in the VRd‐S group, whereas the POU family (POU2F2/POU2F3/POU5F1B/POU3F4/POU2F1) was enriched in the VRd‐R group (Figure 6G). In brief, footprinting was used to call the differential motif binding of POU2F2 and RUNX1 within promoters, and we found that the eccDNA binding activity of POU2F2 was significantly decreased in the VRd‐S group and that the motif activity in the V/Rd‐R and VRd‐R groups was also more significant than that in the VRd‐S group (Figure 6H). Survival analysis was performed among the POU and RUNX gene families, and POU2F2, RUNX1, and RUNX2 were negatively associated with PFS and OS in the CoMMpass‐VRd cohort (Figure 6I–K). Super‐enhancers (SEs) and typical‐enhancers (TEs) are two genomic loci classes regulating cell identity genes. We thus used ROSE to identify SEs and TEs based on H3K27ac signals across 10 MMCLs (Figure 6L; Figure S7A, Supporting Information). Interestingly, the key VRd resistance‐associated TF genes *POU2F2*, *RUNX1*, and *RUNX2* presented significantly higher expression levels in SEs than in TEs. Hence, we concluded that eccANKRD28 interacted with POU2F2‐RUNX1/2 to initiate enhancer effects and drove MM's aberrant gene regulatory program.

### 2.7 POU2F2‐RUNX1/2‐Based Network Regulates VRd‐Associated Cancer Epigenetics

**Figure 7:**
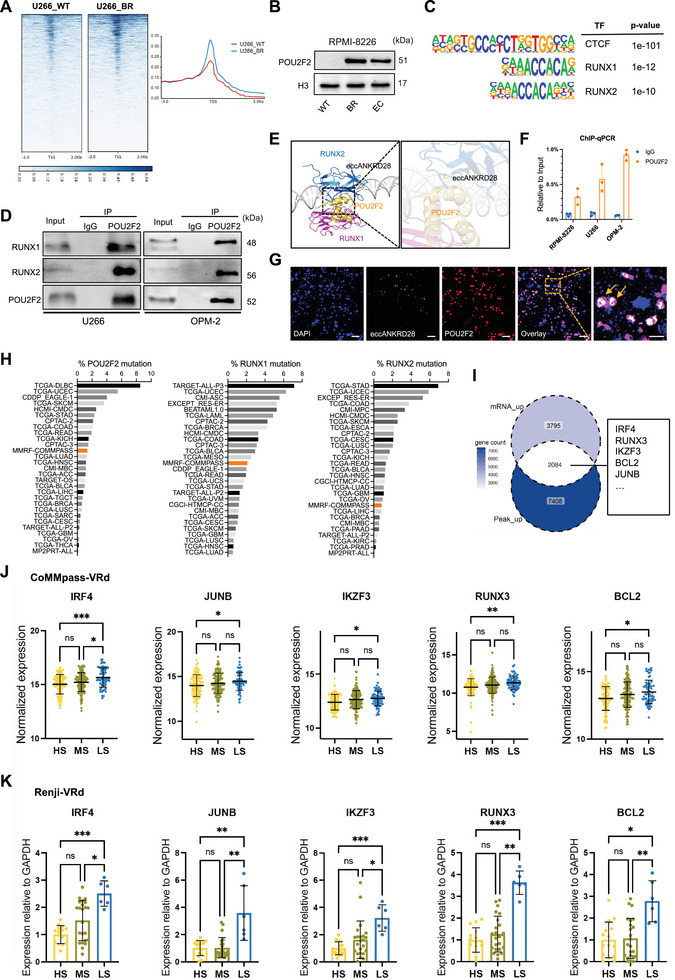
The POU2F2‐RUNX1/2 transcriptional network regulates VRd‐associated cancer epigenetics. A) Left: Heatmap of normalized CUT&Tag tag densities for POU2F2 binding sites called from U266‐WT and U266‐BR cells. Right: The Histogram showing the average tag density of CUT&Tag peaks is displayed below the heatmaps. B) Immunoblotting analysis of POU2F2 in RPMI‐8226‐WT, BR, and EC cells. C) Motif analysis revealed that the CTCF, RUNX1, and RUNX2 binding sites are within the POU2F2 locus. D) The immunoprecipitation was performed using the anti‐POU2F2 antibody in U266 and OPM‐2 cells and then blotted for the indicated proteins. E) Predicted interaction of protein structures of POU2F2 with RUNX1/2 and eccANKRD28. The docking module highlights the hydrogen bonds (yellow) connecting POU2F2 and eccANKRD28. F) ChIP‒qPCR validation of eccANKRD28 binding with the POU2F2 protein in RPMI‐8226, U266, and OPM‐2 cells. G) Representative dual immunofluorescence‐FISH microscopy of eccANKRD28 (green) and POU2F2 (red) colocalization in U266 cells. Box (yellow) is magnified (right). Scale bar, 10 µm. H) POU2F2, RUNX1, and RUNX2 mutation frequency in TCGA and MMRF‐CoMMpass. I) Venn diagrams showing the overlap of differentially up‐regulation genes in U266‐BR cells compared to U266‐WT cells through CUT&Tag and RNA‐seq datasets. J‐K) Oncogene expressions in the CoMMpass‐VRd cohort (J, n = 312) and Renji‐VRd cohort (K, n = 98), separately, grouped by individuals with VRd high sensitivity (HS), median sensitivity (MS, achieving VGPR) and low sensitivity (LS). ^*^ *P* < 0.05; ^**^ *P* < 0.01; ^***^ *P* < 0.001.

POU2F2 (POU class 2 homeobox 2), also known as OCT2, is widely expressed in B cells and B‐cell lineage tumor cells but has not been previously studied in MM.^[^ ^31^, ^32^ ^]^ To further elucidate the potential functional significance of POU2F2, we next performed a CUT&Tag assay. As expected, *POU2F2* upregulated its downstream RNA level directly through RNA‐seq and CUT&Tag chromatin profiling, and POU2F2 peak levels were increased near transcription start sites (TSSs) in U266‐BR cells compared to U266‐WT cells (**Figure** **7**A; Figure S7B, Supporting Information). In line with our CUT&Tag results, the POU2F2 protein level was also significantly increased in the BR cell lines RPMI‐8226 and U266 (Figure 7B; Figure S7C, Supporting Information). To elucidate the mechanism of POU2F2 in VRd resistance, the target genes that POU2F2 may directly regulate were analyzed via macs2. Motif analysis for POU2F2‐bound peaks shows that under the influence of the insulator CTCF, a strong enrichment was recognized by RUNX1 and RUNX2 transcription factor motifs (Figure 7C). To validate their interaction, we conducted the coimmunoprecipitation (Co‐IP) assay. POU2F2 revealed a pull‐down of RUNX1 and RUNX2 proteins in U266 and OPM‐2 cells (Figure 7D). In other words, POU2F2 interacted with RUNX1/2 to form the protein complex. Subsequently, we sought to decipher the structural basis of the POU2F2–eccANKRD28 interaction. As shown in Figure 7E, the sequence‐specific POU2F2‐eccDNA interaction is mediated by numerous base pair‐specific hydrogen bonds. We conducted ChIP‐qPCR (Figure 7F) and dual immunofluorescence‐FISH (Figure 7G) experiments to verify the molecular docking result and found POU2F2 could bind to enhancer eccANKRD28 with a strong relationship of colocalization. Further, analysis of sequencing data from MMRF‐CoMMpass found that *POU2F2*, *RUNX1*, and *RUNX2 gene* mutations were relatively rare (1.36%, 2.09%, and 0.83%, respectively) among MM patients compared to other tumor types profiled in TCGA, suggesting that their functions are preserved in MM (Figure 7H). To probe the molecular mechanism of VRd resistance, we next set out the target up‐regulation genes of the network of TFs downstream of POU2F2‐RUNX1/2. By integrating CUT&Tag and RNA‐seq datasets, we identified the activation and enhanced transcription of multiple oncogenes, including *IRF4*, *RUNX3, IKZF3, BCL2*, and *JUNB* (Figure 7I; Figure S7D, Supporting Information), which were known to be related to drug resistance and validated in the CoMMpass‐VRd dataset (Figure 7J) as well as Renji‐VRd cohort (Figure 7K). Our findings showed that the enhancer eccANKRD28 could bind to POU2F2, which directly acts on RUNX1 and RUNX2 and their downstream drug resistance‐related oncogenes, forming a cascade transcriptional regulatory network.

### 2.8 Enhancer EccANKRD28 Accelerates Tumor Growth and Mediates Drug Resistance in MM Xenograft Models

**Figure 8:**
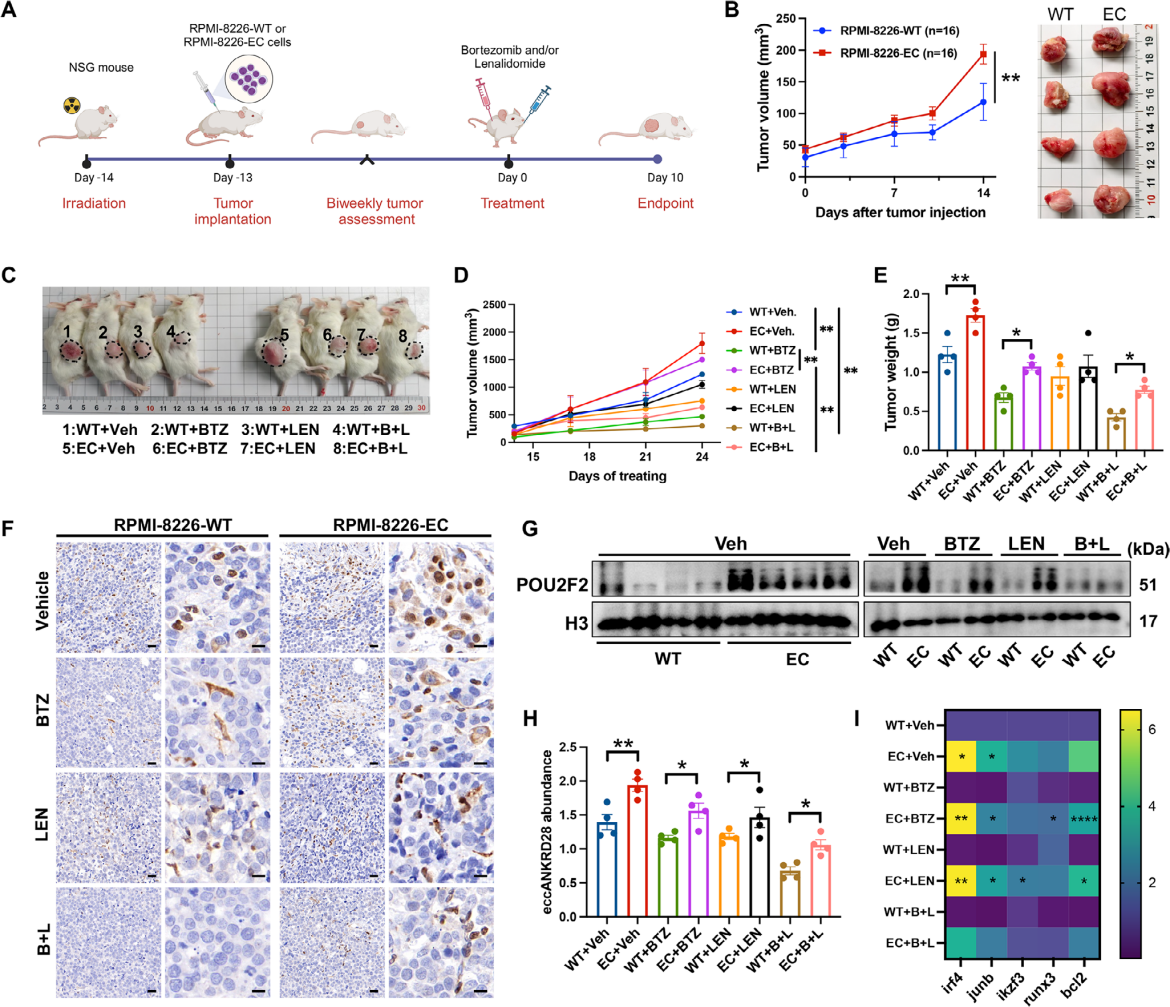
Enhancer eccANKRD28 promotes tumor progression and desensitizes MM cells to VRd in vivo. A) Overview of the eccANKRD28 function in mediating drug resistance using MM subcutaneous xenograft model. The graphical scheme was created with BioRender. B) Images of subcutaneous tumors formed by RPMI‐8226‐WT or RPMI‐8226‐EC cells (n = 16 per group). C) Tumor volume assessments among the eight groups of mice at the endpoint. The groups of 1 to 8 are the group of RPMI‐8226‐WT+Vehicle (WT+Veh), RPMI‐8226‐WT+bortezomib (WT+BTZ), RPMI‐8226‐WT+ lenalidomide (WT+LEN), RPMI‐8226‐WT+bortezomib+lenalidomide (WT+B+L), RPMI‐8226‐EC+Vehicle (EC+Veh), RPMI‐8226‐EC+bortezomib (EC+BTZ), RPMI‐8226‐EC+lenalidomide (EC+LEN), and RPMI‐8226‐EC+bortezomib+lenalidomide (EC+B+L), respectively (n = 4 per group). D) The tumor volume of each group was measured twice a week. E) Tumor weights were analyzed at the endpoint. F) Representative IHC analyses for POU2F2 in subcutaneous tumors (scale, 10 µm). G) Immunoblot illustrating levels of POU2F2 in eccANKRD28 xenografts with vehicle or indicated treatments. H) The eccANKRD28 abundance in mice serum was calculated with qPCR. I) Heatmap of oncogene mRNA expressions in eccANKRD28 xenografts calculated with RT‐qPCR. The oncogene mRNA levels were first normalized to the GAPDH control and then compared to wild‐type (WT) levels, which were arbitrarily set at 1.0. The data from RT‐qPCR analysis were presented as mean ± SD of three individual experiments, and the data from animal experiments were presented as mean ± SEM. Statistical analysis was performed using a two‐way ANOVA.Veh, vehicle; BTZ, bortezomib; LEN, lenalidomide; B+L, bortezomib+lenalidomide. ^*^ *P* < 0.05, ^**^ *P* < 0.01, ^****^ *P* < 0.0001.

It was also interesting to validate the ability of eccANKRD28 manipulated MM cells to enhance drug resistance to bortezomib, lenalidomide, and their combination in vivo. Therefore, we used immunodeficient mice and RPMI‐8226‐WT/EC cells to develop a xenograft model. Two weeks after inoculation, these mice were randomized to treatment with intraperitoneal injections of vehicle or bortezomib, oral gavage of lenalidomide, or a combination of both (**Figure** **8**A). As shown in Figure 8B, eccANKRD28 manipulated MM cells could effectively promote the growth of xenograft tumors compared with the parental cells. We subsequently tested if a higher abundance of eccANKRD28 could induce drug resistance to bortezomib or lenalidomide. Notably, tumor acceleration was observed in all animals injected with RPMI‐8226‐EC cells. Furthermore, the following treatments on tumor‐bearing mice demonstrated that eccANKRD28 could desensitize RPMI‐8226 cells to bortezomib. Although lenalidomide alone did not induce tumor growth delay, bortezomib alone did show some activity in this setting (Figure 8C,D), and the combination of both significantly enhanced the therapeutic effects of suppressing tumor growth compared to either agent alone (Figure 8D,E). The histopathological evaluation confirmed the upregulation of POU2F2 in RPMI‐8226‐EC manipulated tumors and indicated regimens mediated target protein degradation to some degree (Figure 8F). Western blot analysis further supported the ability of eccANKRD28 to induce resistance, with a marked increasing level of POU2F2 compared with parental cells (Figure 8G). Increased copy numbers of eccANKRD28 were also observed in mice serum from MM‐bearing mice injected with RPMI‐8226‐EC cells with indicated regimens, including vehicle, bortezomib, lenalidomide, or a combination of both (Figure 8H). Similarly, RPMI‐8226‐EC manipulated xenografts displayed higher mRNA expression of irf4, junb, ikzf3, runx3, and bcl2 than RPMI‐8226‐WT MM‐bearing mice (Figure 8I). Taken together, the above results strongly suggest that eccANKRD28 could desensitize human MM cells to bortezomib/lenalidomide and promote tumor growth.

## 3 Discussion

**Figure 9:**
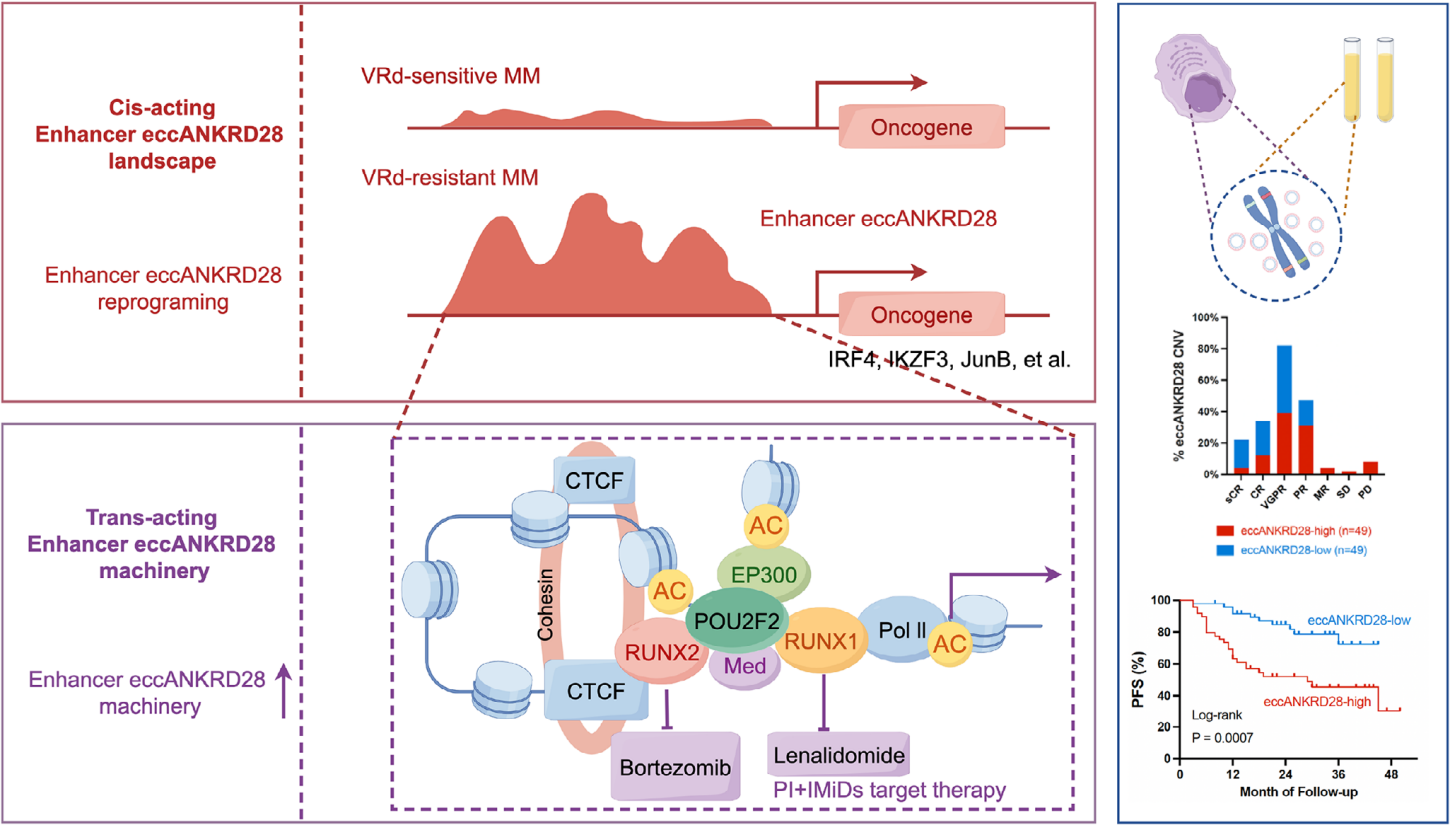
Schematic representation for the role of eccANKRD28 in VRd‐resistant MM cells (created with FigDraw).

Drug resistance is the most important problem in cancer treatment. Genetic abnormalities are important initiation factors for the development of MM, and the role of epigenetic changes in drug resistance has received increasing attention. Here, we report for the first time that enhancer eccANKRD28 manipulated MM cells have been demonstrated to facilitate drug resistance and promote MM progression both in vitro and in vivo. The POU2F2‐RUNX1/2 transcription factor cascade elucidates the epigenetic regulatory basis through which the enhancer eccANKRD28 induces VRd resistance (summarized in **Figure** **9**). These findings hold significant promise for developing critical prognostic and therapeutic clinical tools.

The POU Class 2 homeobox 2 (POU2F2)‐encoded protein binds the octamer sequence 5′‐ATTTGCAT‐3′, a common transcription factor‐binding site in immunoglobulin gene promoters. Since *NF‐κB* activity can also contribute to developing chemotherapy resistance, inhibiting *NF‐κB* is a potential strategy for overcoming chemotherapy resistance in MM.^[^ ^33^ ^]^ Prior research has demonstrated that *NF‐κB* directly activates *POU2F2* transcription in other cancer cells, thus leading to POU2F2 overexpression.^[^ ^34^, ^35^ ^]^ Its expression and binding activity to cis‐regulatory elements are associated with decreased patient survival.^[^ ^36^ ^]^ Bortezomib mainly inhibits the activity of *NF‐kB* through the ubiquitin‐proteasome system (UPS), thus exerting an anti‐MM effect. Our observations indicated a correlation between bortezomib sensitivity and POU2F2 activity, suggesting that POU2F2 is a second target of this medication. Furthermore, the activity of the POU2F2 pathway may serve as a biomarker for predicting the therapeutic efficacy of bortezomib and other drugs that inhibit *NF‐κB*. Ikaros family zinc fingers (IKZFs) can form a trimeric complex with the RUNX1‐binding partner CBFβ, suggesting that RUNX1 and RUNX3 desensitize MM to lenalidomide via protection of IKZFs from degradation.^[^ ^30^ ^]^ *IRF4* orchestrates oncogenic transcription in MM. The IMiDs, such as lenalidomide, can indirectly reduce *IRF4* expression through CRBN‐dependent degradation of *IKZF1* and *IKZF3*.^[^ ^37^, ^38^ ^]^ Myeloma cell‐derived RUNX2 promotes myeloma progression,^[^ ^39^ ^]^ and the hypermethylation or alteration of RUNX2 contributes to B‐cell lineage resistance.^[^ ^40^, ^41^ ^]^ *JUNB* can protect MM cells against Vd‐induced cell death.^[^ ^42^ ^]^ *BCL2* is a common, highly expressed prosurvival (antiapoptotic) protein in neoplastic plasma cells.^[^ ^43^ ^]^ In summary, activating the transcription of these oncogenes directly regulated by POU2F2 results in drug resistance. The CCCTC‐binding factor (CTCF) functions as a versatile transcriptional regulator capable of binding to boundary elements and facilitating the establishment of chromatin loops in conjunction with cohesin proteins. CTCF is also necessary for the maintenance of enhancer‐centric and *NF‐κB*‐dependent chromatin interactions.^[^ ^44^ ^]^ The predominant genomic sites where CTCF binds in MM cells are in intergenic regions, with some sites overlapping with transcriptional enhancers.

To improve the therapeutic efficacy of PI or IMiDs, endogenous DNA generation inhibition should be first prioritized. Environmental stress causes genomic instability and drives local chromosomal breakage, leading to eccDNA formation by self‐joining DNA segments.^[^ ^45^, ^46^ ^]^ In the absence of centromeres, eccDNA elements are inherited unequally, contributing to intratumoral heterogeneity and conferring a proliferative or survival advantage to cancer cells under drug pressure. Following this idea, we used CRISPR/Cas9 to generate a genic and intergenic eccDNA, eccANKRD28, to improve our understanding of the role of eccANKRD28 in drug resistance, which may facilitate the modeling of human genetic disorders caused by eccDNA. Both cHR and NHEJ participate in eccDNA genesis and the suppression of eccDNA exclusion by fostering cell cycle arrest at the G2/M checkpoint.^[^ ^47^ ^]^ Inhibition of cHR or NHEJ activity may reduce the copy number of eccDNA. In addition, eccDNA‐based strategies can disturb the binding of eccDNA, disrupt critical TF‐eccDNA interactions, and restrict binding at the epigenetic level by modulating chromatin accessibility. Approaches to directly target TFs, such as POU2F2, RUNX1, and RUNX2, include inhibiting their expression (RNA interference or miRNAs). They are among the most promising anti‐MM strategies with a potentially high therapeutic index. However, large surface areas for TF‒DNA interactions, which are difficult to target, and predominant nuclear localization, which makes TFs less accessible to therapeutic agents, remain challenging.

Although our NSG xenograft MM mouse model demonstrated that eccANKRD28‐manipulated MM cells mediate drug resistance in vivo, this approach has limitations compared to the Cre‐loxP system. A recent study successfully utilized the Cre‐loxP system to simulate ecDNA generation and its role in mouse tumorigenesis; however, this approach is currently limited to large ecDNA fragments (>1 Mbp).^[^ ^48^ ^]^ No established system can safely and stably introduce and maintain specific small eccDNA fragments (such as the 213 bp eccANKRD28 in our study) into animal models via gene editing. Additionally, the NSG model lacks a functional immune system, failing to recapitulate the complex tumor microenvironment (TME) critical for drug resistance. The TME, including immune cells, stromal cells, and extracellular matrix components, plays a critical role in tumor progression and drug resistance. In contrast, the Cre‐loxP model allows for conditional manipulation of eccANKRD28 in specific cell types within an intact immune system, providing a more physiologically relevant context. Differences in drug metabolism between NSG mice and humans may also affect the interpretation of drug resistance mechanisms.

The overexpression of POU2F2 is correlated with aggressive cancer phenotypes and unfavorable prognoses across various malignancies. Consequently, our observations could apply to other hematologic malignancies, including DLBCL and AML, characterized by chromosome instability.^[^ ^49^ ^]^ We highlighted that serum eccDNA could predict the treatment response, which makes them ideal candidates for non‐invasive biopsy biomarkers. Nonetheless, it is essential to recognize the constraints due to this research's relatively modest sample size. Our findings thus require further investigation and validation in larger cohorts with uniform treatment, as eccANKRD28 has the potential to introduce a minimally invasive drug sensitivity methodology to identify individuals with VRd‐resistance and POU2F2‐RUNX1/2 transcriptional cascade‐targeted therapies in patients with high eccANKRD28 copy number. Our study highlights how integrative multi‐omic analysis can be applied to explore and identify novel eccDNA‐oriented targets against multi‐drug resistance from an epigenetic perspective.

## 4 Experimental Section

### Human Samples

A total of 108 serum samples from 10 healthy donors and 98 NDMM patients were analyzed in this study, and the clinical information was described in Table S1 (Supporting Information). Briefly, serum samples were obtained before administering three cycles of the VRd regimen. Afterward, their therapeutic responses were evaluated. Peripheral blood samples were collected and centrifuged at 1600 × g for 10 min at 4 °C. The serum portion was centrifuged at 16000 × g for 10 min at 4 °C to remove residual cells and debris. Three distinct groups were selected from the cohort: healthy donors (HD, n = 10) and NDMM patients who either responded well (achieving sCR/CR, high sensitivity, HS, n = 28) or poorly (achieving PR/MR/SD/PD, low sensitivity, LS, n = 30) to VRd treatment. Five individuals per group were subsequently used for the discovery cohort (NDMM1‐10) via Circle‐seq and RNA‐seq. Another five individuals per group were used for the validation cohort (NDMM11‐20) via qPCR and Sanger‐seq, and the clinical information of these two cohorts was described in Table S2 (Supporting Information). The remaining samples were used in subsequent experiments, especially for copy number quantification. All experiments of candidate eccDNA function in this study comparing the HS and LS groups were carried out in separate cohorts. Among the 98 NDMM patients, 84 were enrolled in the clinical trial, which was registered at www.chictr.org.cn as #ChiCTR2000036105, and the other 14 were retrospectively registered. The cohort was selected to assess the response to the VRd regimen in NDMM patients and to screen for associated biomarkers for more accurate prediction. Written informed consent was obtained from all participants. Patient clinical response was established according to the IMWG guidelines,^[^ ^50^, ^51^ ^]^ and cytogenetic risk classifications were based on the Mayo Stratification of Myeloma and Risk‐Adapted Therapy (mSMART) criteria.^[^ ^52^ ^]^

### Cell Culture and Reagents

The human MM cell lines RPMI‐8226, U266, and OPM‐2 were purchased from the American Type Culture Collection (ATCC, Manassas, VA). All MM cell lines were maintained in RPMI‐1640 medium (#C11875500BT, Gibco, Suzhou, China) supplemented with 10% FBS (#F0193, Sigma‐Aldrich, Missouri, USA) and 1x penicillin‐streptomycin (P/S) (#15140‐122, Gibco). C4‐2B and HEK293T cell lines were provided by Dr. Chen Yang (Fudan University, Shanghai, China) and maintained in DMEM medium ((#C11995500BT, Gibco) supplemented with 10% FBS and 1x P/S. KaryoMAX Colcemid (#15212012) was purchased from Gibco (Grand Island, NY, USA). Bortezomib and lenalidomide were provided by Chia Tai Tianqing Pharmaceutica Group Co. (Nanjing, China).

Mouse monoclonal anti‐POU2F2 (F‐5, #sc‐377475X), anti‐RUNX1 (A‐2, #sc‐365644), and anti‐RUNX2 (F‐2, #sc‐390351) were purchased from Santa Cruz Biotechnology (CA, USA). Rabbit monoclonal anti‐POU2F2 (#ab179808) antibody was purchased from Abcam (Cambridge, UK). Anti‐H3K27ac (#A2771) antibody was obtained from Abclonal Technology (Wuhan, China). Anti‐gamma H2AX (#9718T) was purchased from Cell Signaling Technology (Beverly, USA). Anti‐histone H3 (#06‐599) was obtained from Sigma–Aldrich (St. Louis, MO, USA). Mouse (#LF101) or rabbit (#LF102) secondary HRP‐conjugated antibodies, RIPA lysis buffer (#PC101), and IP/Co‐IP lysis buffer (#PC105) were purchased from Epizyme Biotech (Shanghai, China). The mouse IgG antibody (#B900620) was from Proteintech (Wuhan, China).

### Serum EccDNA Purification, Circle‐Seq, and AFM Characterization

The purification and enrichment of eccDNA from human serum were carried out as described previously with minor modifications.^[^ ^53^ ^]^ Briefly, serum DNA extraction was performed via a QIAamp Circulating Nucleic Acid Kit (#55114, Qiagen). DNA was digested with an exonuclease (Plasmid‐Safe ATP‐dependent DNase, #E3101K, Epicentre) at 37 °C for 5 min to remove linear DNA, followed by column purification using QIAquick PCR Purification Kit (#28104, Qiagen). The complete removal of linear DNA was confirmed by PCR of the *COX5B* gene (Figure S2D, Supporting Information). The enriched circular DNA was subjected to library preparation with a GenSeq Rapid DNA Lib Prep Kit (#GS‐LC‐004, GenSeq Inc, China). Sequencing was performed on an Illumina NovaSeq 6000 with 150 bp paired‐end mode. Atomic force microscopy (AFM) measurements were performed with Dimension icon ScanAsyst (Bruker, USA).

### Bulk RNA‐Seq

Total RNA was used to remove the rRNAs with a GenSeq rRNA Removal Kit (#GS‐LC‐010, GenSeq Inc). According to the manufacturer's instructions, the rRNA‐depleted samples were subsequently subjected to library construction with the GenSeq Low Input RNA Library Prep Kit (#GS‐LC‐032, GenSeq Inc). Libraries were controlled for quality and quantified via the BioAnalyzer 2100 system (Agilent Technologies, Santa Clara, CA, USA). Library sequencing was performed on an Illumina NovaSeq instrument with 150 bp paired‐end reads.

### MM Cell EccDNA Purification

The cells were suspended in L1 solution (Plasmid Mini AX, #01050, A&A Biotechnology) supplemented with proteinase K (#25530049, ThermoFisher Scientific, USA) before incubation overnight at 50 °C with agitation. After lysis, the samples were treated with an alkaline solution, followed by the precipitation of proteins and the separation of chromosomal DNA from circular DNA through an ion exchange membrane column. Column‐purified DNA was treated with FastDigest MssI (#FD1344, ThermoFisher Scientific) to remove mitochondrial circular DNA and incubated at 37 °C for 16 h. The remaining linear DNA was removed by exonuclease (#E3101K, Epicentre) at 37 °C in a heating block, and the enzyme reaction was carried out continuously for 1 week, with additional ATP and DNase added every 24 h (30 units per day) according to the manufacturer's protocol. EccDNA‐enriched samples were used as templates for phi29 polymerase amplification reactions (REPLI‐g Midi Kit, #150043, Qiagen), which amplified eccDNA at 30 °C for 2 days, followed by purification using QIAquick PCR Purification Kit (#28104, Qiagen).

### Establishing VRd‐Resistant Cell Lines

Cells were grown in their respective culture media and passaged for at least two generations after thawing to ensure proper viability. The parental drug‐naive cells were imitated by 5 nm bortezomib and/or 5 µm lenalidomide and enhanced by doubled dosage every 1 month up to 6 months total. The acquisition of a resistant phenotype was monitored and confirmed by calculating the IC_50_ using the CCK‐8 assay. Cells with IC_50_ over five times were kept for further experiments. The resistant RPMI‐8226 cells reached a maximum concentration of 30.28 nm bortezomib and 55.20 µm lenalidomide tolerance, the U266 cells reached a maximum periodic dose of 16.79 nm bortezomib, and the OPM‐2 cells’ most significant sustainable periodic dose of bortezomib was 57.19 nm (Figure S4B, Supporting Information). All cells were tested negatively for mycoplasma contamination, and STR was authenticated if cultured over 6 months.

### Outward PCR and Inward PCR

Outward directing PCR oligos were designed in Primer3web (v4.0) and devised to yield products across the junctions of 10 detected circular DNA structures (Tables S3,S4, Supporting Information). Each 10 µL PCR mixture typically included 120 ng of phi29‐amplified template (2 µL), 10 µm primer, and 2 × Phanta Max Master Mix (5 µL), and the reaction conditions were 10 min at 95 °C followed by 40 cycles of 15 sec at 95 °C and 60 sec at 60 °C. Eight serial dilutions of purified human plasmid DNA were used to produce standard curves for each run, and eccDNA copy numbers were calculated. Concentrations of standards were measured using the Qubit dsDNA High Sensitivity Assay (#32854, ThermoFisher Scientific). Inward‐designed oligos (Table S5, Supporting Information) represent positive controls for PCRs with circular and linear DNA templates. Size separation of the PCR products via 2% agarose gel electrophoresis and Sanger sequencing confirmed the circular structure of the selected eccDNAs. The median copy number was used as a cutoff to categorize high and low eccANKRD28.

### Quantitative RT–PCR (qRT‒PCR)

Total RNA was extracted using TRIzol (Cat#15596018, Invitrogen, USA) and reverse‐transcribed into complementary DNA (cDNA) with PrimeScript RT Master Mix (#RR036A, Takara, Tokyo, Japan). qRT‒PCR was carried out using a CFX96 real‐time PCR instrument (Bio‐Rad, California, USA) and TB Green *Premix Ex Taq* II (#RR820A, Takara) according to manufacturer instructions. A melting curve was created for each amplicon to verify its accuracy. The levels of target mRNAs were normalized to those of *GAPDH*.

### Metaphase Chromosome Spreads

Chromosome spreads were obtained by dropping mitotic cells on glass slides. Briefly, cells were concentrated in metaphase by treatment with 100 ng mL^−1^ KaryoMAX Colcemid (#15212012, Gibco, USA) for 3 h and then incubated overnight. The cells were washed once with PBS, and a single‐cell suspension was incubated in 75 mm KCl for 15 min at 37 °C. Cells were then fixed with Carnoy's fixative (3:1 methanol: acetic acid) and spun down. Cells were washed with fixative three additional times. Cells were then dropped onto humidified glass slides.

### DNA Fluorescence In Situ Hybridization (FISH)

CD138+ plasma cell or CD138‐ BM cell fractions were isolated using magnetic‐activated cell sorting technology (#130051301, Miltenyi Biotec, Auburn, CA). For interphase FISH, cells were directly harvested, washed with PBS, and fixed using Carnoy's fixative. The green SweAMI‐FISH probe (Servicebio, Wuhan, China) was specially designed for eccANKRD28 (5′‐ATTTATTTACTTATTTAGATAATTATATGTTACACACATT‐3′). Briefly, slides were denatured at 85 °C for 10 min, then the FISH probe was diluted in hybridization buffer, added to the sample, and hybridized at 40 °C overnight in a humid and dark chamber. Slides were then washed with 2 × SSC, 1 × SSC, and 0.5 × SSC at 37 °C for 5 min each. Drop the pre‐warmed secondary probe mixture and hybridize at 40 °C for 45 min. Slides were then washed successively with pre‐warmed 2 × SSC, 1 × SSC, 0.5 × SSC, and 0.1 × SSC at 40 °C for 5 min each. Add pre‐warmed signal probe mixture and hybridize at 40 °C for 45 min and then wash again with pre‐warmed 2 × SSC, 1 × SSC, 0.5 × SSC, 0.1 × SSC for 5 min each. For dual immunofluorescence‐FISH, slides were followed by blocking with 3% BSA, and primary antibodies against H3K27ac (#A2771, Abclonal) or γH2AX (#9718T, Cell Signaling Technology) were added at a final dilution of 1:100 followed by overnight incubation at 4 °C. The secondary antibody goat anti‐rabbit Cy3 (#A0516, Beyotime) was added at a final dilution of 1:200 for 1 h at room temperature. The slides were washed and mounted with DAPI. Images were captured under a confocal microscope (Olympus, FV3000, Japan).

### CUT&Tag Assay

U266‐WT and U266‐BR cells were harvested, counted, and centrifuged at 600 × g for 5 min at room temperature. ≈100 000 cells per sample were subjected to CUT&Tag as previously described.^[^ ^54^ ^]^ Briefly, the cells were bound to Concanavalin A‐coated magnetic beads, and the cell membrane was permeabilized with digitonin. The enzyme pA‐Tn5 transposase precisely binds the DNA sequence near the target protein under POU2F2 antibody (#ab179808, Abcam) guidance and results in factor‐targeted tagmentation. The DNA sequence was tagmented, with adapters added simultaneously at both ends, which could be enriched by PCR to form sequencing‐ready libraries. After PCR, the libraries were purified with AMPure beads, and library quality was assessed on an Agilent Bioanalyzer 2100 system. The library preparations were sequenced on an Illumina NovaSeq platform at Tianjin Novogene Bioinformatic Technology Co., Ltd. (Beijing, China), and 150 bp paired‐end reads were generated.

### ChIP‒qPCR Assay

A SimpleChIP Plus Enzymatic ChIP Kit (#9003, Cell Signaling Technology) was used to detect the ability of H3K27ac or POU2F2 to recruit the eccANKRD28 fragment. The cells were fixed with formaldehyde and then lysed, and the chromatin was partially digested with microcecal nuclease to form fragments. The chromatin fractions were incubated with one of the following antibodies: H3K27ac (#A2771, Abclonal) or normal rabbit IgG (#4620, Cell Signaling Technology), POU2F2 (#sc‐377475X, Santa Cruz Biotechnology) or normal mouse IgG (#B900620, Proteintech) with ChIP‐grade protein G magnetic beads. After protein‐DNA was decrosslinked, purification was performed using DNA purification centrifuge columns, and enrichment of eccANKRD28 or oncogenes was detected by qPCR. The qPCR system consisted of nuclease‐free H_2_O, 5 µm primer, and SimpleChIP universal qPCR premix. A total of 40 cycles of the standard PCR program were performed according to the manufacturer's protocol. The signal obtained from each immunoprecipitation was expressed as a percentage of the total input chromatin: % of input = 1%× 2 (Ct 1% input sample−Ct IP sample), Ct = Threshold cycle of the PCR. The qPCR primers used for the ChIP assay are listed in Table S6 (Supporting Information).

### Cell Viability

MM cell viability was measured using a CCK‐8 assay kit (Dojindo, Japan) following the manufacturer's instructions. The transfected cells were seeded into culture plates, and the incubated cells were treated with 10 µL of CCK‐8 reagent. The absorbance was measured at 450 nm. IC_50_ values were derived by a dose‐response inhibition curve and were fitted using nonlinear regression.

### Immunoblotting and Coimmunoprecipitation (Co‐IP)

All procedures followed the standard protocol previously reported.^[^ ^15^ ^]^ The U266 and OPM‐2 cell lines were used for Co‐IP analysis. Cells were lysed in lysis buffer for 30 min on ice. The lysates were incubated overnight at 4 °C on a rotator with 5 µg of anti‐POU2F2 (#sc‐377475X, Santa Cruz Biotechnology) and mouse IgG antibodies (#B900620, Proteintech). 30 µL of protein A/G magnetic beads (#HY‐K0202, MCE, USA) were transferred to the protein‐antibody complexes. Immunoprecipitates were collected after 2 h incubation. PBST was used to wash the pellet fraction four times before resuspending it in 50 µL of 1 × SDS‐PAGE loading buffer and western blotting with monoclonal antibodies against POU2F2 (#179808, Abcam), RUNX1 (#sc‐365644, Santa Cruz Biotechnology), and RUNX2 (#sc‐390351, Santa Cruz Biotechnology), histone H3(#06‐599, Sigma–Aldrich).

### Soft Agar Clonogenicity Assay

RPMI‐8226, U266, and OPM‐2 cells were harvested after 180 days of treatment with dimethylsulfoxide (DMSO), bortezomib, or lenalidomide and seeded into a 6‐well plate at 1000 cells per well. After 24 h, the cells from each condition were treated with either DMSO, 50 nm bortezomib, or 5 µm lenalidomide over 20 days in triplicate. At 20 d, the cell culture medium was aspirated; the cells were washed gently with PBS, fixed in 4% PFA in PBS for 20 min, stained with 1% crystal violet, washed once with PBS, and dried for 30 min. The area intensity was calculated using ImageJ (NIH, USA). Three independent biological replicates were run for each cell line.

### CRISPR/Cas9 Editing

The construction of the RPMI‐8226‐CRISPR/Cas9‐eccDNA (RPMI‐8226‐EC) cell line was conducted through CRISPR–cas9‐mediated approaches with minor modifications.^[^ ^26^ ^]^ The insertion region was selected for the eccANKRD28 interval chr3:15768491‐15768703 (213 bp). The eccDNA sequences were retrieved from the UCSC Genome Brower, and repetitive and low‐complexity DNA sequences were annotated and masked by RepeatMasker. The guide sequences of sgRNAs were designed with the online software tool CRISPRCasFinder (Table S7, Supporting Information). The guide sequence selected was constructed into U6‐CMV‐mCherry‐P2A‐puro‐WPRE. EccANKRD28 guide and control RNA lentiviral particles were produced by transfecting HEK293T cells together with packaging plasmids using the jetPRIME transfection reagent (#101000046, Polyplus, Illkirch, France) according to the manufacturer's instructions and following the standard lentivirus production protocol.^[^ ^55^ ^]^ The medium was replaced with RPMI medium without additives 4 h post‐transfection, and medium containing virus particles were collected 48 and 72 h post‐transfection. RPMI‐8226 cells with eccANKRD28 region were infected with lentivirus; 3 days after infection, puromycin (0.75ug mL^−1^) was added to the culture medium for 48 h. One week after transfection, the puromycin‐resistant cells were harvested, and mCherry+ single cell was sorted using a BD FACS Arial III and cultured in 96‐well until 70–80% confluence. Single clones typically evolve after 21 days in culture. The stable clones were further expanded, and their genomic DNA (gDNA) was extracted and subjected to genotyping with a pair of primers flanking the eccDNA region. The PCR product of the genotyping results was subjected to Sanger sequencing to confirm the insertion at the predicted cutting site. Establishing the stable RPMI‐8226‐CRISPR/Cas9‐eccDNA cell line could be validated by imaging eccANKRD28 with metaphase FISH, and RT‐qPCR assessed the copy number of the generated eccANKRD28.

### Xenograft Experiments

All animal experimental protocols were approved by the Medical Experimental Animal Care Commission of Renji Hospital (RJ2025‐007B). The investigation was conducted according to the ethical standards, the national and international guidelines, and the Committee for Ethical Review of Research Involving Animal Subjects at Renji Hospital (approval number: RJ2025‐007B). 2 × 10^6^ RPMI‐8226‐WT or RPMI‐8226‐EC cells were injected subcutaneously into 6‐week‐old female NSG mice (strain: NOD.*Cg‐Prkdc^scid^Il2rg^em1Smoc^*, n = 16 mice per group). At day 14 after tumor establishment, the RPMI‐8226‐WT/EC tumor‐bearing mice were randomized into four groups [vehicle, bortezomib (0.5 mg kg^−1^), lenalidomide (30 mg kg^−1^), or combination of both. n = 4 mice per group], and treatments were started. Bortezomib (0.5 mg kg^−1^) was administrated intraperitoneally three times per week, while lenalidomide (30 mg kg^−1^ in 0.9% CMC‐Na) was administered daily via oral gavage with 0.9% CMC‐Na as vehicle control. Tumor growth was monitored twice a week by measuring tumor size in two orthogonal dimensions, and tumor volume was calculated by the following equation: tumor volume = (length × width^2)/2. After 10 days of treatment, the mice were euthanized, and the tumors were isolated and weighed. The tumors were then divided into two parts, one fixed in formalin for immunohistochemistry staining and the other stored in liquid nitrogen for immunoblotting and RNA exaction.

### Immunohistochemistry (IHC)

Tumor xenografts derived from in vivo mouse models were fixed in formalin for 24 h. Microarray slides were cut at 4 microns on plus slides. Slides were allowed to air dry. Slides were placed in a 55–60 °C oven for 30 min. The IHC stains were performed on the Ventana automated immunostainer at 37 °C. Tissue sections were incubated with anti‐POU2F2 (#sc‐377475X, Santa Cruz Biotechnology) at a final dilution of 1:200 overnight at 4 °C. The evaluation of stained proteins was performed by assessing both the intensity (scored as 0, 1, 2, or 3) and the extent of staining (scored as 0, 0%; 1, <10%; 2, 10–50%; 3, >50%) as previously described.^[^ ^56^ ^]^

### Protein–DNA Interaction Docking Study

Protein structures were obtained from PDB (http://www.rcsb.org/; POU2F2: 1HDP, RUNX1: 3WTS, RUNX2: 6VG8). Docking of protein‐protein interactions was performed using ClusPro (https://cluspro.org/), and protein‐DNA docking was conducted using HDOCK (http://hdock.phys.hust.edu.cn/). PyMOL was used to generate 3D illustrations.

### CUT&Tag/ChIP‐Seq Data Analysis

CUT&Tag/ChIP‐seq data analysis was performed as previously described with slight modifications.^[^ ^57^, ^58^ ^]^ In summary, raw data in fastq format were first processed using fastp. Clean reads were mapped to the human genome version hg38 via Bowtie2. Only uniquely mapped (MAPQ ≥ 13) and deduplicated reads were used for further analysis. All peak calling was performed with macs2 using “macs2 ‐q 0.05 ‐f AUTO –call‐summits –nomodel –shift ‐100 –extsize 200 –keep‐dup all”. Peaks with a q‐value threshold of 0.05 were used for all the datasets by default. Peaks were adjusted to the exact size‐centered peak sums, and motif discoveries of these loci sequences were performed using findMotifsGenome.pl program in HOMER v4.11 software with “‐len 8,10,12,14 ‐gc ‐size given ‐homer2 ‐dumpFasta”. ChIPseeker was used to retrieve the nearest genes around the peak and annotate the genomic region of the peak.^[^ ^59^ ^]^ Peaks of different groups were merged via “bedtools merge”. The mean RPM of each group in the merged peak was calculated. Only peaks with a fold change in the RPM greater than 2 were considered differential. Responses associated with different peaks were identified using ChIPseeker. Heatmaps were plotted using the computeMatrix and plotHeatmap commands in deepTools.^[^ ^60^ ^]^ The normalized CUT&Tag‐seq data were visualized using the Integrative Genomics Viewer (IGV).

### scATAC‐Seq and scRNA‐Seq Analysis

scATAC‐seq and scRNA‐seq downstream were reanalyzed with Seurat and ArchR, respectively.^[^ ^3^, ^61^ ^]^ All cells with a total number of <3000 fragments, a TSS enrichment score <7, a doublet‐enrichment score >6, and a predicted doublet score >100 were excluded (Figure S6, Supporting Information). Myeloma cells from all patients were normalized, and the dimensionality was reduced via iterative latent semantic indexing and clustering. Peak calling was performed with macs2 motif deviations calculated via the JASPAR database. For each TF motif, a mean value was calculated.

### Statistical Analysis

Statistical analysis used GraphPad Prism 10, RStudio (version 4.2.2), and Python 3. The RESOURCES TABLES in Supporting Information could provide details of the software used. Survival probabilities were estimated through the Kaplan‒Meier method. For mutation analyses, for a given gene, the somatic mutation frequency in each TCGA and the MMRF‐CoMMpass cohort (the unique mutated cases/total unique cases in the cohort) were calculated using the GDC Mutation Frequency Tool provided on the GDC portal (https://portal.gdc.cancer.gov/). Two‐way ANOVA was used to calculate the difference in IC50 with various treatments. The significance of the variations was assessed using either the Kruskal‒Wallis test or the Student's t‐test. All p values reported were two‐tailed, with a threshold of P < 0.05 deemed statistically significant across all analyses. Data were presented as mean ± SD in cell experiments and mean ± SEM in animal studies, ^*^ *P* < 0.05, ^**^ *P* < 0.01, ^***^ *P* < 0.001, ^****^ *P* < 0.0001, and all above experiments have been repeated three times. All analyses were performed using the human genome reference sequence GRh38. Detailed information regarding the statistical tests employed for each experiment can be found in the corresponding figure legends.^[^ ^62^, ^63^ ^]^

### Ethical Statement

The study was approved by the Ethics Committee of Renji Hospital, Shanghai Jiao Tong University School of Medicine (Approval No: SK2020‐086) and conducted following the Declaration of Helsinki.

## Conflict of Interest

The authors declare no conflict of interest.

## Author Contributions

B.C. and J.H. conceived and designed the project. J.H. supervised the research. B.C. wrote the manuscript, performed all the experiments and/or bioinformatics analysis. Y.Z. performed the Circle‐seq analysis. B.C. and C.S. performed the CRISPR/Cas9 cell line editing. L.N., T.L. and J.H. gave advice and revised the manuscript. F.X., L.Z., M.Z and H.H. recruited and followed up with patients. J.L., D.Z. and G.Z. collected samples. All authors approved the final manuscript.

## Supporting information



Supporting Information

## Data Availability

The datasets are available in the following repository: (i) The raw sequence data of Circle‐seq and RNA‐seq reported in this paper have been deposited in the Genome Sequence Archive in National Genomics Data Center, China National Center for Bioinformation/Beijing Institute of Genomics, Chinese Academy of Sciences (GSA‐Human: HRA008118) that are publicly accessible at https://ngdc.cncb.ac.cn/gsa‐human. (ii) POU2F2 CUT&Tag and RNA‐seq data are deposited under HRA008134 and HRA008136. (iii) scATAC‐seq and scRNA‐seq data have been published previously and deposited at the European Genome‐phenome Archive under the accession numbers EGAS00001006538 and EGAS00001004805 (https://www.ebi.ac.uk/ega/). (iv) Previously published H3K27 acetylation ChIP‐seq data are available from the European Nucleotide Archive under accession code PRJEB25605. (v) RNA‐seq data (n=312) from the BMPCs of VRd‐treated NDMM patients enrolled in the CoMMpass study (version IA11) were obtained from MMRF.
