# Supplementary material for: Enhancer Extrachromosomal Circular DNA ANKRD28 Elicits Drug Resistance via POU2F2‐Mediated Transcriptional Network in Multiple Myeloma
Source: Adv Sci (Weinh). 2025 Apr 1;12(21):2415695. doi: 10.1002/advs.202415695 (PMC12140331; doi:10.1002/advs.202415695)
Supplement: Supplementary file 1 — Supporting Information [file ADVS-12-2415695-s001.docx]

Supplementary Tables

**Table S1. Characteristics of NDMM patients and donors at sampling.**

| Parameter |  | \| **NDMM Patients** \|  \| \| --- \| --- \| | Healthy Donors |
| --- | --- | --- | --- | --- | --- |
| Patients, n |  | 98 | 10 |
| Gender, n (%) | Male | 46 (47) | 5 (50) |
|  | Female | 52 (53) | 5 (50) |
| Age at diagnosis, years (range) |  | 62 (44-78) | 61 (52-73) |
| ISS Stage, n (%) | I | 43 (44) | NA |
|  | II | 33 (34) | NA |
|  | III | 22 (22) | NA |
| R-ISS Stage, n (%) | I | 39 (40) | NA |
|  | II | 50 (51) | NA |
|  | III | 9 (9) | NA |
| Chromosomal abnormalities, n (%) | Del (13q) | 41 (42) | NA |
|  | Gain (1q) | 41 (42) | NA |
|  | Del (17p) | 7 (7) | NA |
|  | t (11;14) | 21 (21) | NA |
|  | t (4;14) | 12 (12) | NA |
|  | t (14;16) | 3 (3) | NA |
| Cytogenetics, n (%) | High‐risk | 54 (55) | NA |
|  | Standard‐risk | 44 (45) | NA |
| FLCR(*i/u*), n (%) | 0.01-100 | 65 (65) | NA |
|  | ≤0.01, ≥100 | 15 (15) | NA |
|  | NA | 18 (18) | NA |
| β2-MG (mg/L), n (%) | ≥3.5 | 39 (40) | NA |
| BMPC (%), n (%) | >30 | 39 (40) | NA |
|  | ≤30 | 41 (42) | NA |
|  | NA | 18 (18) | NA |
| ALB (g/L), n (%) | <35 | 38 (39) | NA |
| LDH (U/L), n (%) | ≥245 | 13 (13) | NA |
| CRP (mg/L), n (%) | ≥6 | 15 (15) | NA |
| VRd Response, n (%) | sCR/CR | 28 (28) | NA |
|  | PR/MR/SD/PD | 30 (31) | NA |
|  | VGPR | 40 (41) | NA |

**Table S2. Patient Characteristics of the discovery and validation cohort.**

|  | ID | Gender | Age at diagnosis | ISS  Stage | R-ISS  Stage | VRd Response |
| --- | --- | --- | --- | --- | --- | --- |
| Discovery cohort | NDMM1 | M | 49 | II | II | sCR |
|  | NDMM2 | F | 60 | II | II | sCR |
|  | NDMM3 | M | 68 | I | II | sCR |
|  | NDMM4 | M | 57 | III | III | CR |
|  | NDMM5 | F | 53 | III | III | sCR |
|  | NDMM6 | F | 53 | II | II | PR |
|  | NDMM7 | F | 45 | II | II | PR |
|  | NDMM8 | M | 49 | II | II | PR |
|  | NDMM9 | F | 64 | II | II | PR |
|  | NDMM10 | F | 62 | III | III | PR |
| Validation cohort | NDMM11 | M | 70 | II | II | CR |
|  | NDMM12 | F | 55 | I | II | sCR |
|  | NDMM13 | F | 62 | I | I | sCR |
|  | NDMM14 | M | 61 | II | II | sCR |
|  | NDMM15 | F | 72 | II | III | sCR |
|  | NDMM16 | M | 63 | II | III | PD |
|  | NDMM17 | F | 74 | III | III | PD |
|  | NDMM18 | F | 57 | III | III | MR |
|  | NDMM19 | F | 63 | III | II | PR |
|  | NDMM20 | M | 70 | II | II | PR |

**Table S3. Characteristic of 10 candidate eccDNAs.**

| Lane NO. | eccDNA | ecc_chr | ecc_location | ecc_size (bp) | PCR_product (bp) | ORF  (Mini Pep Len=75nt) |
| --- | --- | --- | --- | --- | --- | --- |
| 1 | eccACOT7 | 1p36 | chr1:6276033-6276620 | 588 | 126 | 3 |
| 2 | eccANKRD28 | 3p25.1 | chr3:15768491-15768703 | 213 | 126 | 0 |
| 3 | eccAXIN2 | 17q24.1 | chr17:65538025-65538145 | 121 | 83 | 0 |
| 4 | eccFOXM1 | 12p13 | chr12:2873650-2873998 | 349 | 179 | 0 |
| 5 | eccGNAQ | 9q21 | chr9:77760904-77761134 | 231 | 154 | 2 |
| 6 | eccMLLT3 | 9p22 | chr9:20351509-20352285 | 777 | 246 | 0 |
| 7 | eccMORC1 | 3q13 | chr3:109108218-109109175 | 958 | 196 | 3 |
| 8 | eccNLRP3 | 1q44 | chr1:247438956-247439080 | 125 | 88 | 0 |
| 9 | eccRAD9A | 11q13.1-q13.2 | chr11:67360108-67360458 | 351 | 186 | 0 |
| 10 | eccRASA3 | 13q34 | chr13:114001251-114001365 | 115 | 66 | 0 |

**Table S4. Outward primers of 10 candidate eccDNAs.**

| **eccDNA** | **primer-F** | **primer-R** |
| --- | --- | --- |
| eccACOT7 | AAGAAAGCTGACGTGGGGAT | AGGAGGAGGTGTAGACAGGG |
| eccANKRD28 | GCAACAGAGCAAGATCTTCTC | TCAAGCCATCCTACCTCAGC |
| eccAXIN2 | ACCCTAACGCACCCCATG | CTGCGTGTGGGTGTGTGC |
| eccFOXM1 | TGGGATTACAGGGATGAGCC | CCTGGGAGGTGGAGGTTG |
| eccGNAQ | TGGGAAGTGAGGAGCG | TCAGACGGGGCGGTTG |
| eccMLLT3 | TCTGTCCCACCCTCCTATGA | GGCCCAGGATCACATCTAGG |
| eccMORC1 | GGCTCCCTCCCTTTCATCAT | TTGGGGTTTGTGACTGGAGA |
| eccNLRP3 | TCCATCAATCCATCCAAGGG | TGACGGATGGAAAATAGAGGGA |
| eccRAD9A | GTGGTTCATGCCTGTAATCCA | AATTCTCCCACCTCAGCCTC |
| eccRASA3 | GATCAGGGTGGGCAGTGG | TCCCTGACCCTGAGCCCA |
| pGEX-5X-2 | GGGCTGGCAAGCCACGTTTGGTG | CCGGGAGCTGCATGTGTCAGAGG |

**Table S5. Inward primers of target genes.**

| **Gene** | **Species** | **primer-F** | **primer-R** |
| --- | --- | --- | --- |
| *ANKRD28* | Human | GACGCCTCTGATGCTATCTGTTCTC | TGTCTGCTGTGGCTGGATTTGC |
| *GAPDH* | Human | ACAACTTTGGTATCGTGGAAGG | GCCATCACGCCACAGTTTC |
| *COX5B* | Human | GGGCACCATTTTCCTTGATCAT | AGTCGCCTGCTCTTCATCAG |
| *IRF4* | Human | GCTGATCGACCAGATCGACAG | CGGTTGTAGTCCTGCTTGC |
| *JUNB* | Human | ACGACTCATACACAGCTACGG | GCTCGGTTTCAGGAGTTTGTAGT |
| *IKZF3* | Human | GCTCATACAGACCCGCATGAT | AACTGGAACCATCTCCGAGGT |
| *RUNX3* | Human | GCGAGGGAAGAGTTTCACCC | TTGATGGCTCGGTGGTAGGT |
| *BCL2* | Human | GGTGGGGTCATGTGTGTGG | CGGTTCAGGTACTCAGTCATCC |
| *gapdh* | Mouse | AACTTTGGCATTGTGGAAGGGCTC | TGGAAGAGTGGGAGTTGCTGTTGA |
| *cox5b* | Mouse | GGAAGACCCTAATCTAGTCCCG | GTTGGCCCATCGCTGACTC |
| *irf4* | Mouse | TCCGACAGTGGTIGATCGAC | CCTCACGATTGTAGTCCTGCTT |
| *junb* | Mouse | TCACGACGACTCTTACGCAG | CCTTGAGACCCCGATAGCGA |
| *ikzf3* | Mouse | CTGAATGACTACAGCTTGCCC | GCTCCGGCTTCATAATGTTCT |
| *runx3* | Mouse | CAGGTTCAACGACCTTCGATT | GTGGTAGGTAGCCACTTGGG |
| *bcl2* | Mouse | ATCCCTTTGTGGAACTATATGGC | GGTATCCACCCAGAGTGATGC |

**Table S6. Primers for ChIP-qPCR.**

| **Gene** | **primer-F** | **primer-R** |
| --- | --- | --- |
| *eccANKRD28* | GAGTCCATTGCAGGCATATTCT | CAAACGGCTTAACTGCTTTCGTGTG |
| *GAPDH* | TTTGGTATCGTGGAAGGACTCA | AGGAGCCAGTCTTGGATGAGAA |
| *IRF4* | GGGTTTCTTTCCACGGGACT | GAACAAAAGGTTCCCGCGTC |
| *JUNB* | TTTACAAGGACACGCGCTTC | CTTTCCTGGCGTCGTTTCC |
| *IKZF3* | CTGGAATGGGCGTTCACCTT | TGAGCTTTTCCCCTAGGCATC |
| *RUNX3* | ATGACCTTGGGCTCTGGTTC | GTCTGTGTTCGGAGTCCTGG |
| *BCL2* | CAGCCATGTGGTACGGTTCT | TCCCGCTACACACTCAGAGA |

**Table S7. sgRNAs for CRISPR/Cas9 assay.**

|  | **sgRNA** |
| --- | --- |
| sgRNA1 | ACAGGTGTTCCAGTAAAGGG AGG (target sequence+ PAM) |
| sgRNA2 | GACGTATCTTAACATTTGGG AGG (target sequence+ PAM) |
| sgRNA3 | AAAAGCAGCATTAAAATTAT AGG (target sequence+ PAM) |
| sgRNA4 | GAGGTCTCACTATATTGCCC AGG (target sequence+ PAM) |

RESOURCES TABLES

| **Software and Algorithms** | **Version** |
| --- | --- |
| Seurat | 4.4.0 |
| ArchR | 1.0.1 |
| samtools | 1.9 |
| survival | 3.5-5 |
| survminer | 0.4.9 |
| fastp | 0.20.0 |
| fastqc | 0.11.3 |
| bwa | 0.7.12 |
| deepTools | 3.0.2 |
| macs2 | 2.1.0 |
| ggplot2 | 3.5.0 |
| ggthemes | 5.1.0 |
| bedtools | v2 |
| bowtie2 | 2.3.4.1 |
| BioSeqUtils | 0.0.8 |
| rtracklayer | 1.58.0 |
| PyMOL | 2.4.1 |
| CRISPRCasFinder | 4.2 |

Supplementary Figures

**Figure S1** A) Pie chart represents the proportion of eccDNA overlapped 0, 1, 2, 3, 4, 5, or more than six genes. B) Average length of eccDNAs screened in the serum of HD, HS, and LS patients. Data is shown as the mean ± SD. **P* < 0.05.

**Figure S2** A) Bar plot and split violin plot for validations of the differences in normalized counts of 10 candidate eccDNAs in (left) Circle-seq and (right) qPCR in another 10 NDMM patients (**P* < 0.05; ***P* < 0.01; ****P* < 0.001). B) Outward PCR detection of 10 candidate eccDNAs using purified eccDNA from patients. C) Venn diagram shows significant overlapping eccDNAs among HD, HS, and/or LS patients. The crossed two eccDNAs refer to eccANKRD28 and eccMORC1. D) COX5B gene detection after 5-day exonuclease treatment of HD/HS/LS patients’ serum after column purification (Circle-seq method). N: Negative control; T: No template control; P: Positive control; M: DNA marker. E) (left) Schematic diagram of outward PCR (blue arrows) and inward PCR (black arrows). (right) Junctional sites obtained after Sanger sequencing of PCR products in Figure 2F.

**Figure S3** A) FISH detection of eccANKRD28 in paired CD138+ plasma cells and CD138- cells in bone marrow. Scale bar, 50 μm. B) Mean fluorescent intensity of eccANKRD28 between paired CD138- and CD138+ BM samples were compared and analyzed by Wilcoxon matched-pairs signed rank test (****P* < 0.001). C) Outward PCR detection of eccANKRD28 using genomic DNA from 5 NDMM patients. Total eccDNA served as the positive control sample and GAPDH served as the positive control of genomic DNA. D) Kaplan–Meier curves of PFS (p = 0.031) and OS (p = 0.011) in 312 NDMM patients in the CoMMpass-VRd cohort stratified by eccGene ANKRD28 . P values were derived from a two-sided log-rank test without correction for multiple hypotheses.

**Figure S4.** A) Standard curve construction based on a plasmid with a known copy number using qPCR. B) Dose-response curves for parental (WT) and BR model in U266 and OPM-2 cells.

**Figure S5** A) UCSC genome browser capture showing representative cCREs (putative promoters and enhancers), DNase-seq (open chromatin regions), H3K4me1 (active enhancer marker), H3K27ac and CTCF signals at selected eccANKRD28 locus, chr3: 15768491-15768703, mapping to the human reference genome GRCh38. B) Nuclear staining with DAPI pre- and post-sonication before ChIP-qPCR assay. Scale bar, 20 μm. C) Chromatin was digested to 1 ~ 5 nucleosomes (150 ~ 900 bp) in RPMI-8226, U266, and OPM-2 cells.

**Figure S6** A) Distribution of transcription start site (TSS) enrichment scores and number of unique nuclear fragments in single cells for each patient. B) Violin plot for each sample for (top) the TSS enrichment scores and (bottom) log10(unique nuclear fragments). C) Plot for each sample for (top) fragment size distribution and (bottom) TSS enrichment profiles. D) UMAPs of scATAC-seq data colored by (top) the number of unique nuclear fragments and (bottom) the per-cell TSS enrichment score. Dot color represents the density in arbitrary units (AU) of points in the plot. E) UMAP projection of single cells colored by gene activity scores of *TNFRSF17, SDC1, CD38,* and *ANKRD28* through scATAC-seq.

**Figure S7** A) ROSE-calling using H3K27ac ChIP-seq showed super-enhancers (SEs, red) and typical-enhancers (TEs, blue) in EJM, KMS-12-BM, MM.1S, MOLP-2, NCI-H929, RPMI-8226, MOLP-8 and LP-1. B) Normalized RNA-seq expression and CUT&Tag signals of POU2F2 between U266-WT and U266-BR cells. C) Immunoblotting analysis of POU2F2 in U266-WT and U266-BR cells. D) Details of overlapped and upregulated genes ranking by log2FC in the RNA-seq dataset, including *IRF4, JUNB, IKZF3, RUNX3, and BCL2*.
